# Integrated transcriptome and microRNA analysis reveals molecular responses to high-temperature stress in the liver of American shad (*Alosa sapidissima*)

**DOI:** 10.1186/s12864-024-10567-w

**Published:** 2024-07-01

**Authors:** Ying Liu, Zhengyuan Liang, Yulin Li, Wenbin Zhu, Bingbing Feng, Wei Xu, Jianjun Fu, Panpan Wei, Mingkun Luo, Zaijie Dong

**Affiliations:** 1 Wuxi Fisheries College, Nanjing Agricultural University, Wuxi, Jiangsu China; 2 Key Laboratory of Freshwater Fisheries and Germplasm Resources Utilization, Ministry of Agriculture and Rural Affairs, Freshwater Fisheries Research Center, Chinese Academy of Fishery Sciences, Wuxi, Jiangsu China; 3 Fisheries Technology Extension Center of Jiangsu Province, Nanjing, Jiangsu China; 4 Qinghai Provincial Key Laboratory of Breeding and Protection of Gymnocypris przewalskii, Rescue Center of Qinghai Lake Naked Carp, Xining, Qinghai China

**Keywords:** *Alosa sapidissima*, Heat stress, Liver, Genes, miRNAs

## Abstract

**Background:**

Fish reproduction, development and growth are directly affected by temperature, investigating the regulatory mechanisms behind high temperature stress is helpful to construct a finer molecular network. In this study, we systematically analyzed the transcriptome and miRNA information of American shad (*Alosa sapidissima*) liver tissues at different cultivation temperatures of 24 ℃ (Low), 27 ℃ (Mid) and 30 ℃ (High) based on a high-throughput sequencing platform.

**Results:**

The results showed that there were 1594 differentially expressed genes (DEGs) and 660 differentially expressed miRNAs (DEMs) in the LowLi vs. MidLi comparison group, 473 DEGs and 84 DEMs in the MidLi vs. HighLi group, 914 DEGs and 442 DEMs in the LowLi vs. HighLi group. These included some important genes and miRNAs such as *calr*, *hsp*90*b*1, *hsp*70, ssa-miR-125a-3p, ssa-miR-92b-5p, dre-miR-15a-3p and novel-m1018-5p. The DEGs were mainly enriched in the protein folding, processing and export pathways of the endoplasmic reticulum; the target genes of the DEMs were mainly enriched in the focal adhesion pathway. Furthermore, the association analysis revealed that the key genes were mainly enriched in the metabolic pathway. Interestingly, we found a significant increase in the number of genes and miRNAs involved in the regulation of heat stress during the temperature change from 24 °C to 27 °C. In addition, we examined the tissue expression characteristics of some key genes and miRNAs by qPCR, and found that *calr*, *hsp*90*b*1 and dre-miR-125b-2-3p were significantly highly expressed in the liver at 27 ℃, while novel-m0481-5p, ssa-miR-125a-3p, ssa-miR-92b-5p, dre-miR-15a-3p and novel-m1018-5p had the highest expression in the heart at 30℃. Finally, the quantitative expression trends of 10 randomly selected DEGs and 10 DEMs were consistent with the sequencing data, indicating the reliability of the results.

**Conclusions:**

In summary, this study provides some fundamental data for subsequent in-depth research into the molecular regulatory mechanisms of *A*. *sapidissima* response to heat stress, and for the selective breeding of high temperature tolerant varieties.

**Supplementary Information:**

The online version contains supplementary material available at 10.1186/s12864-024-10567-w.

## Background

The American shad (*Alosa sapidissima*) belongs to the order Clupeiformes, family Clupeidae, genus *Alosa* [1, 2]. At the same time, both *A*. *sapidissima* and the Chinese shad (*Tenualosa reevesii*) are valuable fish subordinated to the shad subfamily that have considerable economic value. *A*. *sapidissima* is famous for its delicacy and has basically the same shape and size as *T. reevesii*. The docosahexaenoic acid (DHA) content and nutritional value of the two are surprisingly the same [2]. By the late 1990s, *T. reevesii* stocks were seriously depleted and the population had declined sharply to the point of near extinction [3]. Meanwhile, China began to import fertilized eggs of *A*. *sapidissima* in the late 20^th^ century, gradually carried out seedling incubation, cultivation, artificial propagation and other works [4]. However, in parallel with the growth and development, both juvenile and adult *A*. *sapidissima* show obvious stress responses to artificial manipulation, water current stimulation and environmental changes, especially temperature changes, which greatly limits the scale and industrialization of shad farming [5].

Temperature, one of the most important environmental factors, influences the growth, metabolism, development, survival and fecundity of fish [6]. *A. sapidissima* is a warm-water fish with a narrow temperature range and low tolerance to high temperature, so high temperature can have a negative effect on its survival. For example, Yuan et al. [7] found that continuous high temperature stress for 96 h significantly inhibited the antioxidant capacity and non-specific immune capacity of *A. sapidissima*, especially the high temperature condition caused inhibition of non-specific immune-related enzyme activities, which might bring about a certain degree of damage to the liver. Yang et al. [8] found that the liver pepsin activity of *A. sapidissima* decreased irreversibly under high temperature stress at 30 °C from 0 to 96 h. Other studies have also confirmed that the external environmental stress such as high temperature can damage normal hepatocytes of the organism, affecting their normal physiological and metabolic functions [9]. Therefore, high temperature is one of the main reasons for the low survival rate of *A. sapidissima* culture.

The liver is an important organ for metabolism which plays an important role in immune defense, detoxification and endocrine hormone synthesis in fish [10]. It is more sensitive to temperature changes and has therefore been used as an object in many studies on fish at high temperature. For instance, Fan et al. [11] conducted stress experiments on the liver of Chinese loach (*Paramisqurnus dabryanus*) in four groups, namely control (22 ℃), high temperature (29 ℃), cooling (15 ℃) and low temperature (8 ℃), the results showed that the activities of liver enzymes, lysozyme (LZM) and alkaline phosphatase (AKP) were significantly affected by the sudden temperature change, the enzyme activities in the experimental groups were significantly lower than those in the control group at the end of the stress period. Alanine aminotransferase (ALT) and aspartate aminotransferase (AST) are two important aminotransferases in fish that can be used as markers of liver damage [12]. Sun et al. [13] domesticated tongue sole (*Cynoglossus semilaevis*) in seawater with a temperature of 17 ℃ for 2 weeks, then directly exposed them to heat shock in seawater with a temperature of 21 ℃ (T21), 24 ℃ (T24) and 27 ℃ (T27) for 1.5 h, then placed them in seawater at 17 ℃ for rearing. The results showed that the ALT level in the T21 and T24 groups decreased to the level of the control group after 48 h, while it was still higher in the T27 group than in the control group after 96 h. AST levels increased in the T27 group (peaked after 6 h), then returned to the level of the control group, while there was no significant change in AST levels in the T21 and T24 groups after stress. These results indicated that heat shock damaged the liver and led to changes in hepatocyte function. However, we find that there is still a lack of information on the transcriptome and miRNAs of *A. sapidissima* under heat stress conditions. Furthermore, there is an urgent need to construct a finer molecular regulatory network for heat stress in fish.

The optimum water temperature for the growth of *A. sapidissima* is 15–25 °C [14], if the temperature is above 30 °C, the fish become manic, then gradually die. In this study, we set 24 ℃ as the optimal water temperature of *A. sapidissima* and 30 ℃ as its highest stress temperature to analyze the expression patterns of key genes and miRNAs in liver tissues under different conditions. The results can provide certain basic information for the regulation mechanism of high temperature stress along with subsequent molecular breeding of *A. sapidissima*.

## Results

### Transcriptome data analysis

**Fig. 1:**
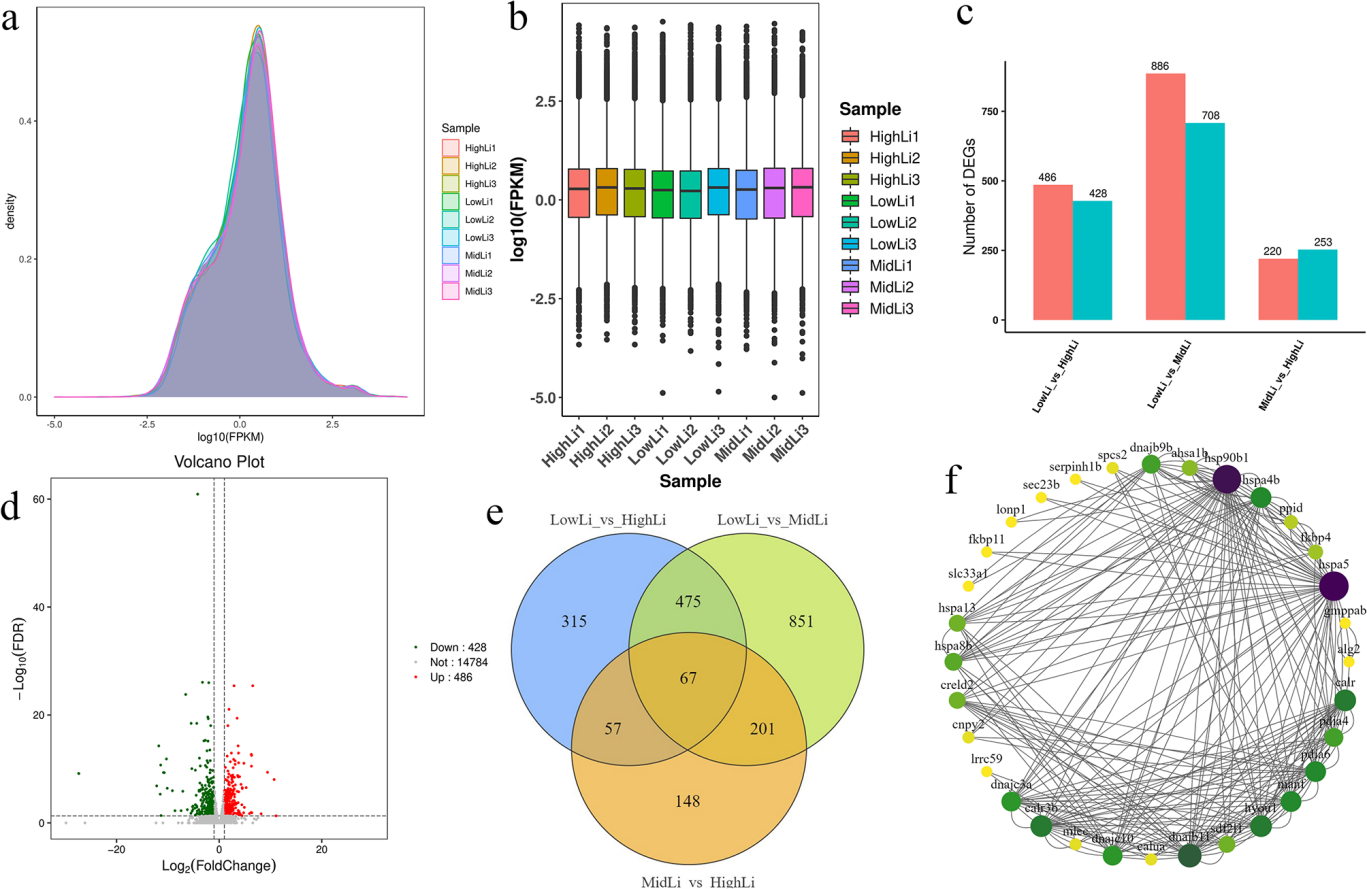
(**a**) Density plot showing the distribution of each sample. (**b**) Expression box-and-line plot. (**c**) Differential gene statistics bar graph. (**d**) Differential expression volcano plot in LowLi vs. HighLi comparison group. (**e**) Differentially expressed gene Venn diagram. (**f**) Protein-protein interaction networks (PPI) interactions of shared differentially expressed genes in the three groups. The color or size of nodes indicates connectivity, purple, green, and yellow indicate high, medium, and low connectivity, respectively, and darker nodes indicate stronger connectivity

A total of 62.84 Gb of transcriptome data was obtained from the 9 samples, of which the percentage of Q30 bases was over 87.88%, indicating high sequencing quality. The matching efficiency of clean reads to reference genome was more than 92.58% (Supplementary file 1). The expression and correlation plots of the samples (Fig. 1a) coupled with the fragments per kilobase of exon model per million mapped fragments (FPKM) values of the samples (Fig. 1b) showed small differences between the biological replicates and a balanced distribution of expression levels. A total of 2981 differentially expressed genes (DEGs) were identified (1592 up-regulated and 1389 down-regulated). The different degrees of heat stress caused a large difference in the number of DEGs between the groups, with the LowLi vs. MidLi group having 1594 DEGs far more than the other two groups, while there were 473 and 914 DEGs in MidLi vs. HighLi group and LowLi vs. HighLi group, respectively (Fig. 1c). The proportion of up-regulated and down-regulated genes in each group was about half and half (Fig. 1d and Supplementary file 5). In addition, Venn plots showed 67 DEGs shared by the three comparison groups (Fig. 1e), indicating their important and conserved roles in high-temperature stress. To analyze the interactions between different genes, we used the STRING protein interaction database for the 67 shared DEGs to better understand the possible regulatory relationships. We found that heat shock protein family A (HSP70) member 5 *(hspa*5), heat shock protein 90 beta family member 1 (*hsp*90*b*1), DnaJ heat shock protein family (HSP40) member B11 (*dnajb*11), hypoxia up-regulated 1 (*hyou*1), cysteine rich with EGF like domains 2 (*creld*2), calreticulin (*calr*), and calreticulin 3b (*calr*3*b*) can interact with multiple genes simultaneously (Fig. 1f), suggesting that they are involved in the regulation of multiple biological functions.

**Fig. 2:**
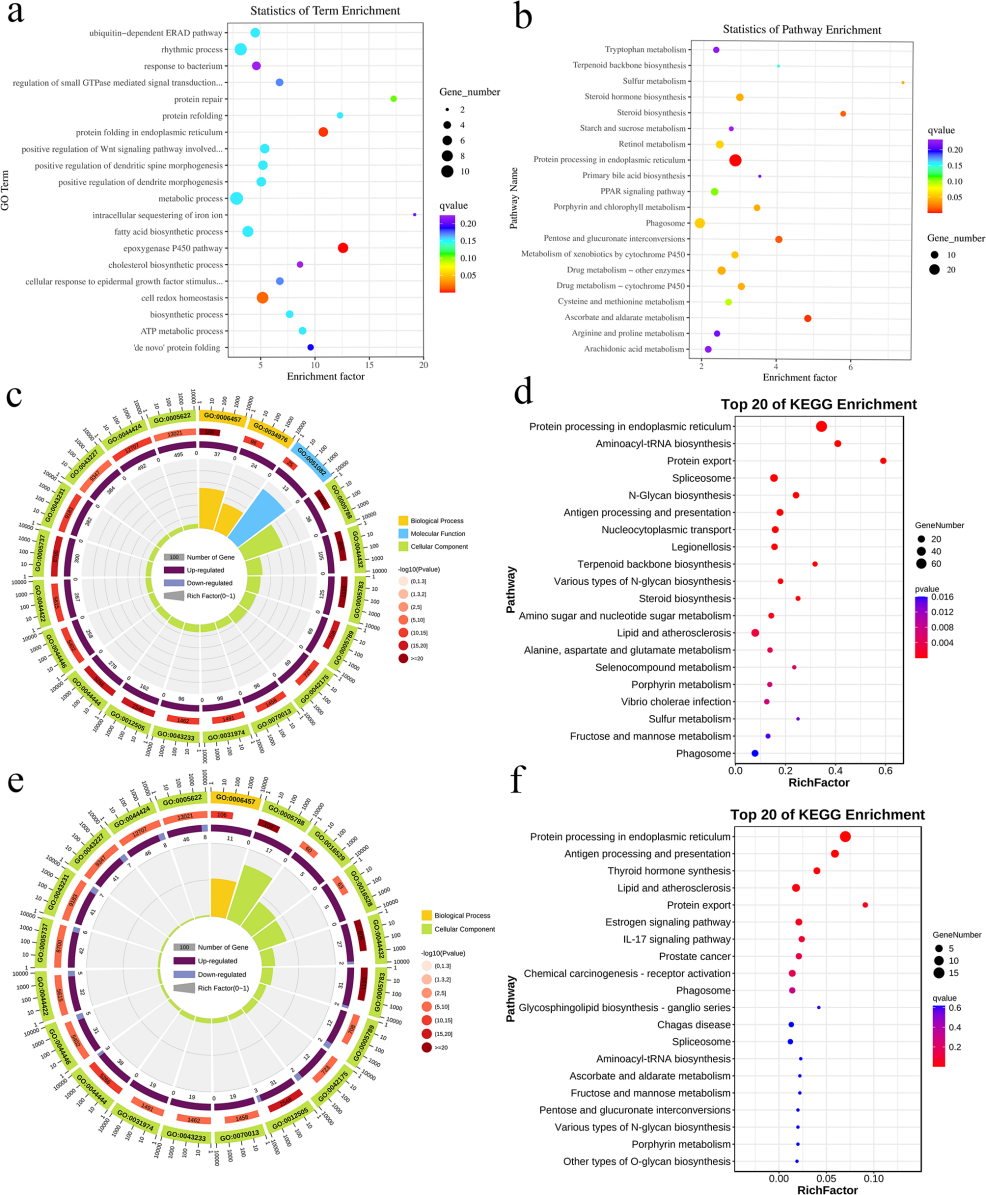
(**a**) Scatter plot of GO pathway enrichment (biological processes) of DEGs in LowLi vs. HighLi group. Where the vertical coordinate is the path and the horizontal coordinate is the enrichment factor. (**b**) KEGG pathway enrichment scatter plot of differentially expressed genes LowLi vs. HighLi group. (**c**) Circle diagram of enrichment of up-regulated genes GO in LowLi vs. MidLi group. The first circle is the top 20 GO term of enrichment, and the outside is the seating scale for the number of DEGs. Different colors represent different ontologies; the second circle is the number of the GO term in the background of DEGs and the Q value. The longer the number, the longer the bar, and the smaller the Q value, the redder the color; the third circle is the proportion of up- regulated and down-regulated DEGs, dark purple and light purple represent the proportion of up- regulated and down-regulated genes, respectively; the fourth circle is the rich factor value of each GO term. (**d**) KEGG pathway enrichment scatter plot for LowLi vs. MidLi up-regulated genes. (**e**) GO enrichment circle plots of shared differentially expressed genes in the three groups. (**f**) KEGG pathway enrichment scatter plot of shared differentially expressed genes in the three groups

We then performed GO and KEGG enrichment analysis for the DEGs in the three groups, the GO enrichment results showed that genes involved in biological processes (BP) such as protein folding, ubiquitin-dependent endoplasmic-reticulum-associated protein degradation (ERAD) pathway, cellular redox homeostasis were significantly enriched in LowLi vs. MidLi and MidLi vs. HighLi groups, genes involved in protein folding in the endoplasmic reticulum was significantly enriched in LowLi vs. HighLi and MidLi vs. HighLi groups. Cellular components (CC) such as endoplasmic reticulum membrane and endoplasmic reticulum lumen were involved in heat stress process. Molecular function (MF) such as oxidoreductase-activating sulfur moiety involved in heat stress process and heat shock protein-binding was significantly enriched in LowLi vs. MidLi and MidLi vs. HighLi. (Fig. 2a and Supplementary file 5). The results of KEGG enrichment showed that DEGs were significantly enriched in the protein processing pathways in the endoplasmic reticulum. Moreover, protein export was found in both the LowLi vs. MidLi and MidLi vs. HighLi comparison groups, which were affected more by heat stress (Fig. 2b and Supplementary file 5).

Additionally, we performed GO and KEGG enrichment of up-regulated and down-regulated genes in the LowLi vs. MidLi, MidLi vs. HighLi and LowLi vs. HighLi comparison groups. The GO enrichment results showed that the genes significantly increased in protein folding and endoplasmic reticulum lumen (Fig. 2c and Supplementary file 5). The KEGG results showed that the up-regulated genes in the LowLi vs. MidLi and LowLi vs. HighLi groups were significantly enriched in the protein processing pathways in the endoplasmic reticulum, with the greatest enrichment in the protein export pathways in the LowLi vs. MidLi (Fig. 2d; Table 1). Besides, we analyzed the 67 shared differential genes for functional enrichment, the GO results showed significant enrichment in protein folding, endoplasmic reticulum, endomembrane system and cytoplasm (Fig. 2e); the KEGG results showed that 16 of the genes were involved in post-translational modifications, protein turnover and acted as molecular chaperones. We also found some genes involved in inorganic ion transport and metabolism, secondary metabolite biosynthesis, nucleotide transport and metabolism, as well as lipid transport and metabolism (Fig. 2f). Moreover, among these 67 shared DEGs, four genes including cerebellin 11 (*cbln*11), *loc*121683193, *loc*121689486 and *loc*121697064 were consistently up-regulated, four genes including *loc*121681730, *loc*121694120, *loc*121699922 and *loc*121712220 were consistently down-regulated with increasing temperature.

**Table 1:** KEGG enrichment of DEGs under heat stress

Category	Description	Count	up-regulation	down-regulation
LowLi vs. HighLi	Protein processing in endoplasmic reticulum	28	28	0
LowLi vs. MidLi	Protein processing in endoplasmic reticulum	75	74	1
MidLi vs. HighLi	Protein processing in endoplasmic reticulum	36	1	35
LowLi vs. MidLi	Protein export	13	13	0
MidLi vs. HighLi	Protein export	4	0	4
LowLi vs. MidLi	Aminoacyl-tRNA biosynthesis	18	18	0
LowLi vs. MidLi	Drug metabolism - cytochrome P450	17	3	14
LowLi vs. HighLi	Steroid hormone biosynthesis	10	2	8
LowLi vs. HighLi	Ascorbate and aldarate metabolism	9	1	8
LowLi vs. HighLi	Pentose and glucuronate interconversions	9	1	8

### miRNAs data analysis

**Fig. 3:**
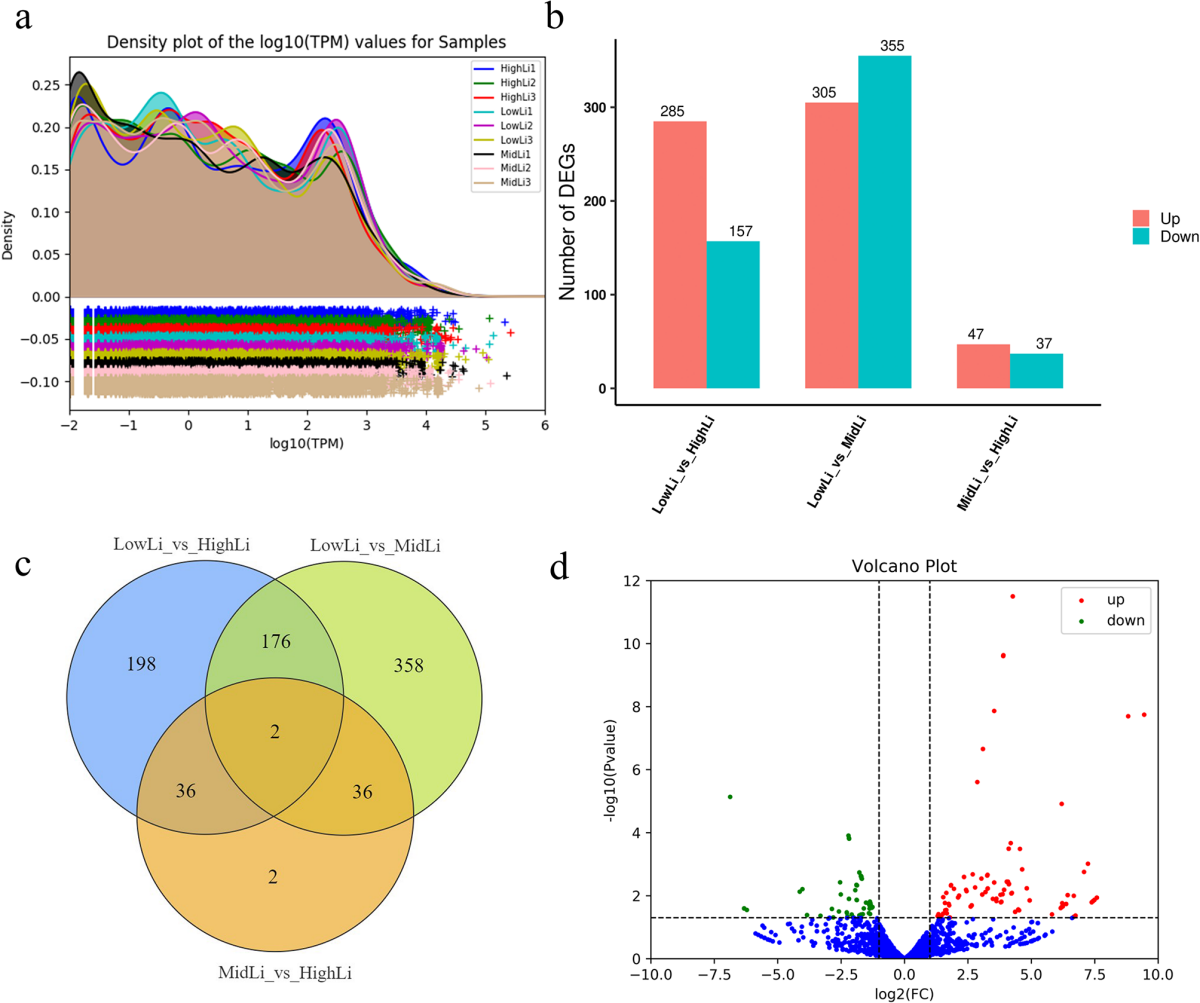
(**a**) TPM density distribution map. The horizontal coordinate represents the logarithmic value of the TPM of the corresponding sample and the vertical coordinate represents the probability density. (**b**) Differential miRNA statistics bar graph. (**c**) Differential expression miRNA Venn diagram. (**d**) Differential expression miRNA volcano plot in LowLi vs. HighLi

As shown in Fig. 3a and Supplementary file 2, the transcripts per kilobase of exon model per million mapped reads (TPM) differences between the biological replicates of each sample were small and reproducible enough to be used in the subsequent analysis. A total of 10,917 (8425 known miRNAs and 2492 novel miRNAs) differentially expressed miRNAs (DEMs) were identified. Of these, 305 significantly up-regulated and 355 significantly down-regulated miRNAs were found in the LowLi vs. MidLi comparison group; 47 significantly up-regulated and 37 significantly down-regulated miRNAs were found in the MidLi vs. HighLi; 285 significantly up-regulated and 157 significantly down-regulated miRNAs were found in the LowLi vs. HighLi group (Fig. 3b); other 2 miRNAs (pma-miR-20a-5p, xtr-miR-106) occurred simultaneously in the three comparison groups, both significantly up-regulated. In addition, there were 38 shared DEMs in the LowLi vs. MidLi and MidLi vs. HighLi comparison groups, 358 DEMs only in LowLi vs. MidLi, 2 DEMs only in MidLi vs. HighLi (Fig. 3c). We also plotted the volcano plot of DEMs in the LowLi vs. HighLi group to visualize the general expression trend (Fig. 3d).

**Fig. 4:**
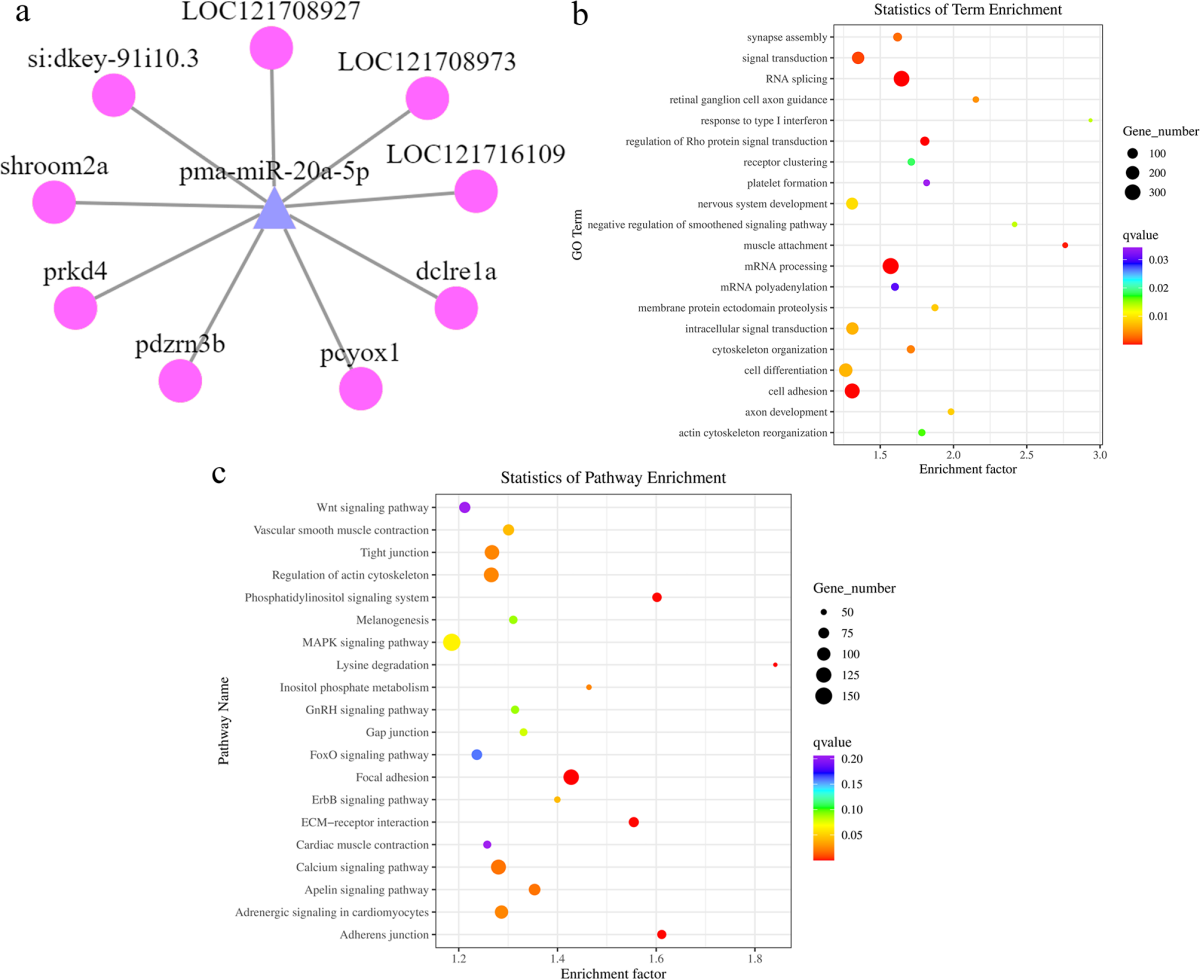
(**a**) miRNA corresponding target gene plots. Circles represent target genes and triangles represent miRNAs. (**b**) Scatter plot of differentially expressed miRNA target genes GO pathway enrichment in LowLi vs. HighLi (biological process). (**c**) KEGG pathway enrichment scatter plot of differentially expressed miRNA target genes in LowLi vs. HighLi

After prediction of target genes, we obtained 1,048,576 mRNA-miRNA interaction pairs (not shown). Among them, 8017 target genes were predicted in the LowLi vs. HighLi comparison group, 10,705 target genes were predicted in the LowLi vs. MidLi comparison group, and 2927 target genes were predicted in the MidLi vs. HighLi group. Moreover, we found that miRNAs and target genes were not in one-to-one relationship during the prediction process, a miRNA can target multiple genes, and at the same time a gene can be regulated by multiple miRNAs, such as pma-miR-20a-5p, which was significantly differentially expressed in the three groups, targeted 9 genes simultaneously (Fig. 4a), whereas the target gene *loc*121718852 was subject to the regulatory effects of 627 miRNAs.

GO analysis revealed that the biological processes mainly involved in all three groups included RNA shearing, mRNA processing and cell adhesion. In addition, the biological processes of transcription and DNA templates were involved in the LowLi vs. MidLi and MidLi vs. HighLi comparison groups. As for cellular components, they were mainly distributed in collagen trimers, the extracellular matrix and other locations. In terms of molecular function, all three groups were mainly involved in metal ion binding, ATP binding, etc. (Fig. 4b and Supplementary file 5). KEGG analysis revealed that these target genes were annotated in numerous signaling pathways, of which, most genes were significantly enriched in the adhesion spot pathway (focal adhesion). In addition, genes in the lysine degradation and Wnt signaling pathways were also significantly enriched in the LowLi vs. HighLi and MidLi vs. HighLi groups (Fig. 4c and Supplementary file 5).

### miRNA-gene regulatory network analysis

Furthermore, we performed association analysis between up-regulated miRNAs and down-regulated expressed genes, as well as between down-expressed miRNAs and up-regulated expressed genes. The results showed 794 pairs of negative correlations between 367 miRNAs and 336 genes in the LowLi vs. MidLi comparison group, 68 pairs of negative regulatory effects between 108 miRNAs and 21 genes in the MidLi vs. HighLi comparison group. Meanwhile, we also found some miRNAs and genes with positive relationships.

### Down miRNA-up gene association analysis

**Fig. 5:**
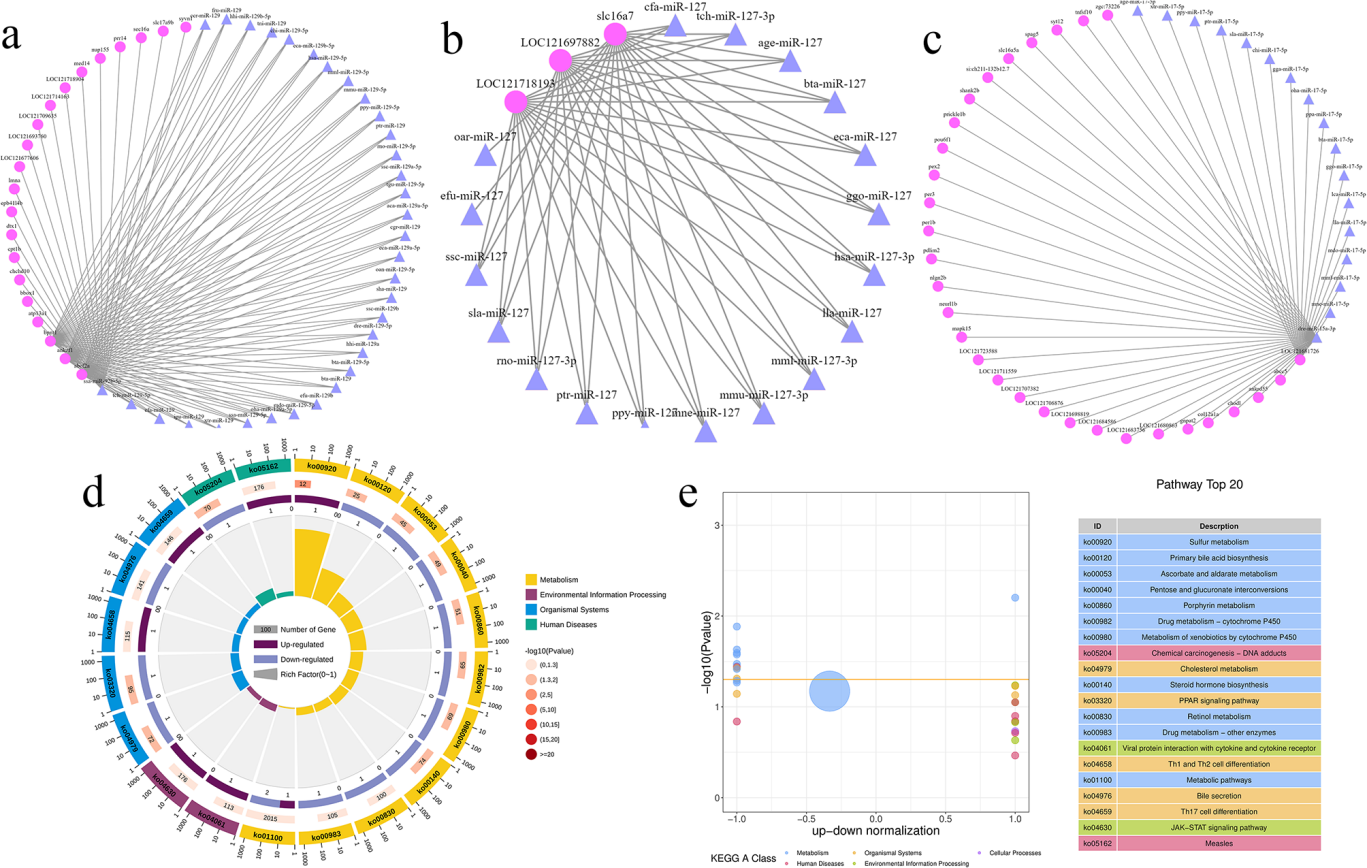
(**a**) Network diagram of down miRNA-up gene interactions in LowLi vs. MidLi. Purple nodes represent miRNAs and red nodes represent genes. (**b**) Network diagram of down miRNA-up gene interactions in MidLi vs. HighLi. Same as above. (**c**) LowLi vs. MidLi up miRNA-down gene interactions network diagram. Same as above. (**d**) Enrichment of key gene pathways in negatively correlated miRNA-gene GO-enriched circle diagram. Same as above. (**e**) KEGG-enriched differential bubble plots of key gene pathways in negatively correlated miRNA-gene. Vertical coordinate is -log10 (*P* value), horizontal coordinate is z-score value, and on the right side is the list of top 20 pathways sorted by *P* value, different colors represent different classifications

The results of the down miRNA-up gene association analysis showed that the major miRNAs and genes in the LowLi vs. MidLi comparison group were ssa-miR-92b-5p, ATP-binding cassette, sub-family F (GCN20), member 2a (*abcf*2*a*), ankyrin repeat and zinc finger peptidyl tRNA hydrolase 1 (*ankzf*1), 3‘(2’), 5’-bisphosphate nucleotidase 1 (*bpnt*1) and others, respectively (Fig. 5a). In the miRNA-gene network of MidLi vs. HighLi, the most important genes were *loc*121718193, *loc*121697882 and solute carrier family 16 member 7 (*slc*16*a*7) (Fig. 5b).

### Up miRNA-down gene association analysis

The association analysis between up-regulated miRNAs and down-regulated expressed genes showed that the major miRNAs and genes in the LowLi vs. MidLi comparison group were dre-miR-15a-3p, novel-m1250-3p, novel-m1425-3p, *si*:*dkey*-91*i*10.3 and *loc*121681726 (Fig. 5c). There was a strong association between novel-m1018-5p and genes such as *loc*121718816 and stabilin 2 (*stab*2) in the MidLi vs. HighLi group.

### Positive correlation association and functional enrichment analysis

There were 61 pairs of down miRNA-down gene and 3 pairs of up miRNA-up gene interactions between 108 miRNAs and 21 genes in MidLi vs. HighLi. The association results showed that the major miRNAs and genes in the positively correlated miRNA-gene network in MidLi vs. HighLi group were efu-miR-127, *loc*121724150, *loc*121688807, and unc-45 myosin chaperone A (*unc*45*a*).

Subsequently, we analyzed the enrichment of key genes in negatively correlated miRNA-genes in the LowLi vs. MidLi and MidLi vs. HighLi groups, our results showed that these key genes were significantly enriched mainly in metabolic pathways such as sulfur metabolism, ascorbic acid and aldose metabolism, porphyrin metabolism, cholesterol metabolism and retinol metabolism (Fig. 5d and e).

### Tissue expression characteristics of key genes and miRNAs

**Fig. 6:**
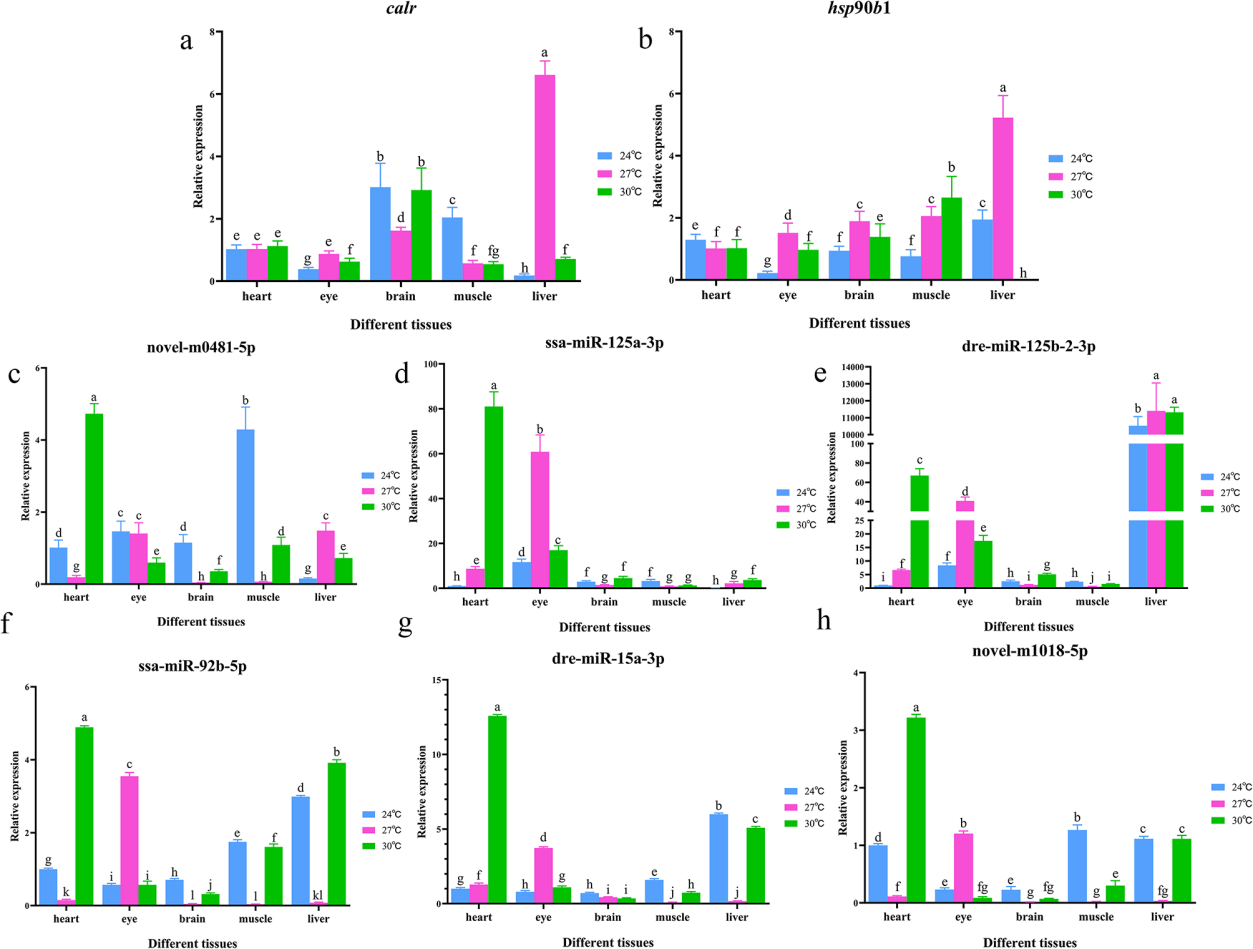
(**a**) Expression of *calr* gene in different tissues at three temperatures. Different letters indicate that the expression of genes or miRNAs in different tissues at three temperatures was significantly different (*P* < 0.05). (**b**) Expression of *hsp*90*b*1 gene in different tissues. Same as above. (**c**) Expression of novel-m0481-5p miRNA in different tissues at three temperatures. Same as above. (**d**) Expression of ssa-miR-125a-3p miRNA in different tissues. Same as above. (**e**) Expression of dre-miR-125b-2-3p miRNA in different tissues. Same as above. (**f**) Expression of ssa-miR-92b-5p miRNA in different tissues. Same as above. (**g**) Expression of dre-miR-15a-3p miRNA in different tissues. Same as above. (**h**) Expression of novel-m1018-5p miRNA in different tissues. Same as above

We selected five different tissues (heart, eye, brain, muscle and liver) to analyze the expression changes of some key genes and miRNAs (including *calr*, *hsp*90*b*1, novel-m0481-5p, ssa-miR-125a-3p, dre-miR-125b-2-3p, ssa-miR-92b-5p, dre-miR-15a-3p and novel-m1018-5p) at three different temperatures. It was found that as temperature increased, the *calr* expression in the eye and liver first increased and then decreased; in the brain it first decreased and then increased; in the muscles, it had a general downward trend (Fig. 6a). However, the *hsp*90*b*1 expression first decreased and then increased in the heart, first increased and then decreased in the eye, brain and liver, gradually increased in the muscle with increasing temperature (Fig. 6b). Meanwhile, we also found that the expression of *calr* and *hsp*90*b*1 was significantly higher in liver tissue at 27 ℃.

On the other hand, along with temperature increased, the expression of novel-m0481-5p in the heart, brain and muscle of *A. sapidissima* initially decreased and then increased; in the liver, its expression initially increased and then decreased; in the eye, its overall decreasing expression trend was observed (Fig. 6c). The expression level of ssa-miR-125a-3p gradually increased in the heart and liver, initially increased and then decreased in the eye, initially decreased and then increased in the brain and muscle (Fig. 6d). The expression of dre-miR-125b-2-3p increased gradually in the heart, increased and then decreased in the eye and liver; initially decreased and then increased in the brain and muscle (Fig. 6e). The expression of ssa-miR-92b-5p and novel-m1018-5p first decreased and then increased in the heart, brain, muscle and liver, while their expressions increased first and then decreased in the eye (Fig. 6f and h). The expression of dre-miR-15a-3p decreased first and then increased in muscle and liver, while it increased first and then decreased in eye, increased overall in heart, and decreased overall in brain (Fig. 6g). In addition, we found that at 30 ℃, novel-m0481-5p, ssa-miR-125a-3p, ssa-miR-92b-5p, dre-miR-15a-3p and novel-m1018-5p had the highest expression levels in the heart; the expression level of dre-miR-125b-2-3p was higher in the liver than that in other tissues at all three temperatures.

### Validation of sequencing results

**Fig. 7:**
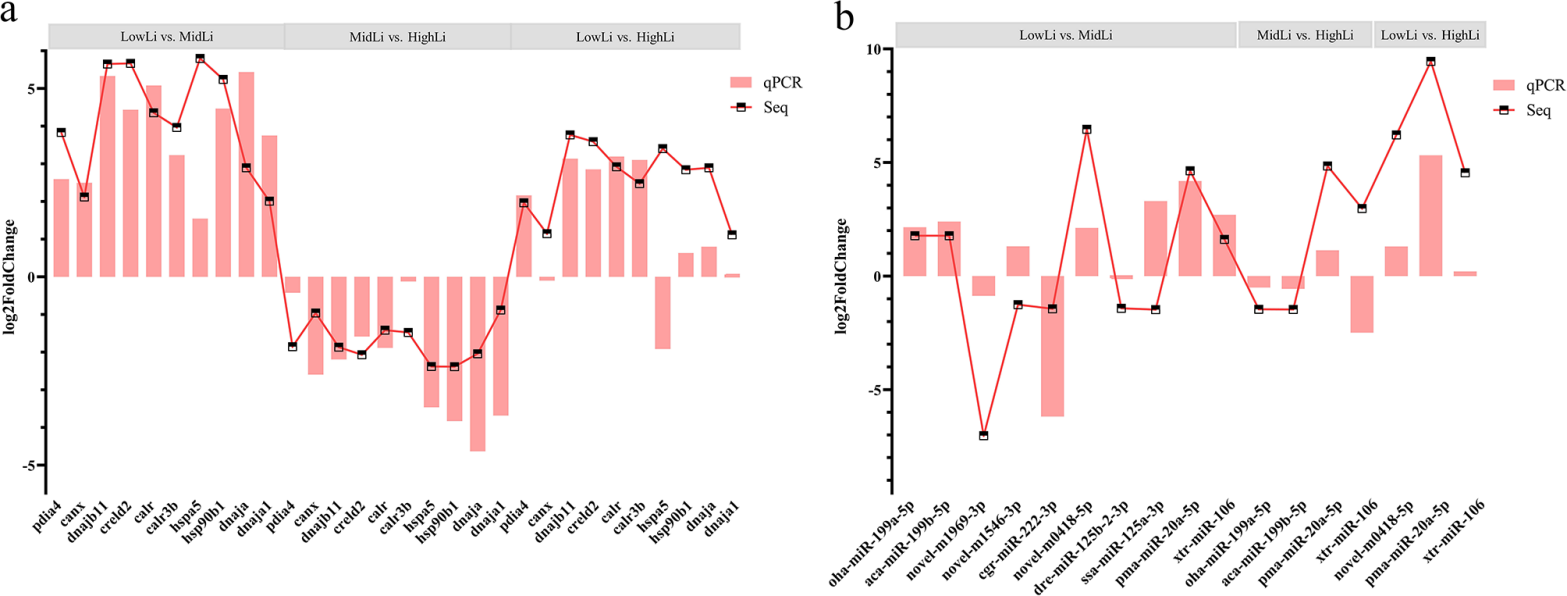
(**a**) The qPCR validation of RNA-Seq at three temperatures. (**b**). The qPCR validation of miRNA-Seq at three temperatures

To verify the accuracy of the high-throughput sequencing results, 10 DEGs and 10 DEMs were randomly selected from all high-throughput sequencing results to check their expression levels in the liver tissue at three temperatures by the qRT-PCR. The results showed that the expression trends of the 10 DEGs and 10 DEMs were basically consistent with those of the sequencing results at the three temperatures, confirming the reliability of the sequencing results (Fig. 7a and b).

## Discussion

In aquaculture, changes in water temperature always have an effect on the physiological condition and behavior of the fish. As one of the organs closely related to changes in the external environment, the liver is more sensitive to changes in water temperature. It has physiological functions such as detoxification, metabolism, synthesis, defense and immunity [10]. Due to the gradual change of the world climate, studies on the environmental adaptability of fish have gradually increased in recent years, some results have shown that “ecological temperature and warmth” is an important factor in the “high temperature hypoxia” of fish [15]. *A*. *sapidissima* is a warm-water fish that is strongly influenced by ambient temperature during development, reproduction and growth. The heat stress alters the structural characteristics of the liver of *A*. *sapidissima*. Luo et al. [16] showed that the *A. sapidissima* hepatocyte damage became serious with increasing temperature, the most pronounced damage occurred at 30 °C. At 24 °C, the liver tissue was structurally clear, whereas as the temperature increased, the tissue showed signs of congestion, vacuolar deformation, blurring of the cellular shape, and lysis of the nuclei. Chronic heat stress often leads to loss of the shad, which severely limits the scale and industrialization of its breeding and aquaculture [5, 6]. In this study, the molecular response in *A. sapidissima* liver coping with chronic heat stress was first investigated using sequencing technology, a large number of heat stress-related genes and miRNAs were identified. The transcriptome results showed there were 914, 1594 and 473 DEGs in the LowLi vs. HighLi, LowLi vs. MidLi and MidLi vs. HighLi comparison groups, respectively (Fig. 1c). Among them, DEGs were significantly increased in the LowLi vs. MidLi comparison group, indicating that more genes involved in the regulation of heat stress during the temperature change from 24 ℃ to 27 ℃, the result was consistent with the previous findings of Luo et al. [16]. GO and KEGG analyses revealed that protein processing and protein export pathways in the endoplasmic reticulum played important roles in the process of heat stress response in *A. sapidissima* (Fig. 2b and Supplementary file 5). In addition, we found that the main function of genes that were constantly up-regulated or down-regulated in response to increasing temperatures was also encoding proteins, of which, the *cbln*11 gene might be related to immunity and defense [17].

Many functions and pathways related to protein folding, processing and export were significantly enriched during heat stress (Fig. 2a, c, d and e and Supplementary file 5), suggesting that protein damage occurred in *A. sapidissima* when temperature increased. Moreover, Wang et al. found that heat led to misfolding of proteins and the accumulation of unfolded proteins, thus triggered stress response in the endoplasmic reticulum to alleviate heat stress [18]. The protein processing pathways in the endoplasmic reticulum was found to be the key pathway involved, and this pathway was significantly enriched in heat stress (Fig. 2a, b and d and Supplementary file 5). In addition, protein-protein interaction (PPI) networks revealed seven shared DEGs could interact with multiple genes simultaneously, including the *hsp* family genes *hspa*5, *hsp*90*b*1, *dnajb*11 and *hyou*1 (Fig. 1f). Heat shock protein (Hsp) is a class of heat stress proteins widely distributed from bacteria to mammals. It is a highly conserved family of proteins that fulfil various functions as molecular chaperones, which play an important role in molecular assembly and protein folding, translocation, maintenance, and repair, removal and degradation of damaged proteins within the cell [19]. In addition, Hsp plays an important role in maintaining protein homeostasis [20]. Heat stress induces the expression of many *hsp* family genes [21], e.g. high temperature stress increased the expression of *hsp*30 and *hsp*70 in Miramichi Atlantic salmon (*Salmo salar*) [22]. In this study, we found that the *hsp* family genes were mainly involved in protein formation, transport and related metabolic processes, suggesting that the up-regulation of their expression was an important strategy for *A. sapidissima* to resist temperature stress. Luo et al. [16] found that Hsp family proteins were significantly up-regulated during heat stress to respond to external temperature changes, which is consistent with our findings. Furthermore, the *hyou*1 gene in the protein processing pathway in the endoplasmic reticulum was significantly enriched in all three comparison groups, through the study of Pan et al. [23] and our results, so we hypothesized that this gene was also closely related to heat stress regulation.

Compared to transcriptome sequencing, small RNA sequencing can help us to understand the complexity and diversity of different intracellular RNA regulatory networks, which is important for the study of biological development, differentiation, metabolism and disease processes [24, 25]. In this study, we found two miRNAs (pma-miR-20a-5p and xtr-miR-106) were involved in regulating the heat stress response in all three comparison groups (Fig. 3c). In other studies, miR-20a-5p was found to inhibit cancer cell proliferation, invasion and migration by down-regulating the expression of homeobox B13 (*hoxb*13) [26, 27], the overexpression of miR-106 promoted cancer cell proliferation and inhibited apoptosis [28, 29]. Furthermore, we found that miRNAs and target genes were not in one-to-one relationship, a miRNA could target multiple genes, and single gene could be regulated by many miRNAs, e.g. pma-miR-20a-5p targeted 9 genes, while the target gene *loc*121718852 was subject to the action of 627 miRNAs. GO and KEGG analyses of the target genes revealed that the biological processes in the three comparison groups mainly included RNA shearing, mRNA processing, cell adhesion and so on, the molecular functions were mainly related to metal ion binding and ATP binding. (Fig. 4b and Supplementary file 5). We also found that the most genes were significantly enriched in the adhesion spot signaling pathway (Fig. 4c and Supplementary file 5). The adhesion spot signaling pathway plays an important role in regulating cell growth, differentiation and migration [30], and is even closely related to the development of many diseases. The lysine degradation signaling pathway and the Wnt signaling pathway were also highly enriched (Fig. 4c and Supplementary file 5). The lysine degradation signaling pathway is related to the maintenance of homeostasis and energy production [31]. The Wnt signaling pathway is a complex network of proteins that promotes the progression of hepatocellular carcinoma [32, 33]. When investigating the reciprocal regulatory networks, we analyzed key genes of the negatively correlated miRNA-gene network in the LowLi vs. MidLi and MidLi vs. HighLi comparison groups, it was found that these genes were significantly enriched in metabolic pathways (Fig. 5d and e), suggesting that liver metabolism was significantly impaired during heat stress. Moreover, key miRNAs in association analysis may be associated with inflammatory damage and cancer (Fig. 5a and c). For example, it was demonstrated that miR-92b-5p inhibitor suppressed interleukin-18 (IL-18) mediated inflammatory amplification after spinal cord injury via interleukin-18-binding protein (IL-18BP) up-regulation [34], miR-15a-3p inhibited proliferation and invasion, promoted apoptosis of cancer cells by regulating tumor protein D52 (*tpd*52) [35, 36].

Besides, heat triggered endoplasmic reticulum stress because the expression of genes related to the protein processing pathways in the endoplasmic reticulum (*calr* and *hsp*90*b*1) in *A. sapidissima* was significantly upregulated in the LowLi vs. MidLi and LowLi vs. HighLi comparison groups. These genes act as endoplasmic reticulum chaperones that can perform protein quality control and maintain endoplasmic reticulum protein homeostasis [37]. *Calr* gene is a highly conserved molecular chaperone protein that is mainly found in the endoplasmic reticulum [38] and plays an important role in cell proliferation, apoptosis and immune response [39]. In this study, we found that *calr* was involved in a variety of cellular processes, including cell adhesion, cell proliferation. *Hsp*90*b*1 gene, a member of the heat shock protein 90 family, encodes a protein localized in the endoplasmic reticulum that is involved in protein folding [40]. It plays a crucial role as a molecular chaperone for oncogenes [41, 42]. Although miRNAs and genes show negative correlation in most cases, there are a few miRNAs and genes that show positive correlation, for instance, miRNA-93 could positively regulate the growth factor receptor bound protein 2 (*grb*2) gene [43]. In this study, both the *calr* and *hsp*90*b*1 genes in the LowLi vs. HighLi and LowLi vs. MidLi comparison groups were targeted by the same miRNA (novel-m0481-5p) and were positively correlated, whereas the *calr* gene in the LowLi vs. MidLi comparison group was also targeted by two other miRNAs (ssa-miR-125a-3p and dre-miR-125b-2-3p) and showed a negative correlation. On this basis, we examined the expression levels of the *calr* and *hsp*90*b*1 genes and the three miRNAs targeting them. The results showed that the *calr*, *hsp*90*b*1 and dre-miR-125b-2-3p had the highest hepatic expression at 27 °C, while novel-m0481-5p and ssa-miR-125a-3p had the highest cardiac expression at 30 °C (Fig. 6). Moreover, key genes in the reciprocal regulatory networks are significantly enriched in metabolic pathways. We hypothesized that the liver tissue was more sensitive when *A. sapidissima* was exposed to external stimuli and that the drastic temperature changes caused metabolic disturbances in the liver tissue. On the other hand, some studies have confirmed that miRNAs play an important role in physiological and pathological processes of the heart, such as cardiac development, cardiac arrhythmia and myocardial injury [44, 45].

## Conclusions

The results showed that high temperature stress affected the expression of genes (e.g. *calr*, *hsp*90*b*1, etc.) and miRNAs (e.g. novel-m0481-5p, etc.) in the liver tissue of *A*. *sapidissima*. The functional enrichment primarily involved in protein processing in the endoplasmic reticulum, protein folding, processing and export pathways. Remarkably, the number of genes related to the regulation of heat stress increased significantly during the temperature change from 24 °C to 27 °C. In addition, liver damage under chronic stress at high temperatures might be associated with inflammation or even carcinogenesis.

## Methods

### Experimental materials

The experimental fish were two-year-old *A*. *sapidissima* obtained by artificial propagation and bred at the Yangzhong base of the Jiangsu Provincial Fisheries Technology Extension Center. The experimental pond was an earthen pond (6666.67 m^2^) used mainly for Chinese mitten crab (*Eriocheir sinensis*) culture, *A. sapidissima* was poly-cultured inside. During the experimental period, water temperature was monitored with a temperature monitoring system. We took tissue samples when the designed “Low” temperature (24.0 ± 0.5 ℃), “Mid” temperature (27.0 ± 0.5 ℃) and “High” temperature (30.0 ± 0.5 ℃) remained stable for three days. The samples for “Low”, “Mid” and “High” temperature were collected on May 20, June 18 and July 21 of 2023, respectively; At the time of sampling, three fish (354 ± 35 g) were randomly selected and anesthetized with phenoxyethanol (0.3 mL/L). The liver was collected and washed with saline, part of the liver tissue was frozen in liquid nitrogen for transcriptome and miRNA sequencing; the remaining part of the liver and other tissues (heart, eyes, brain and muscle) were frozen in liquid nitrogen, then transferred to -80 °C for subsequent analysis.

### Liver transcriptome and miRNA sequencing

The steps of transcriptome and miRNA sequencing were referred to the previous research methods of Luo et al. [46, 47]. Transcriptome sequencing mainly includes data quality control, comparison with the reference genome, transcript assembly, quantification of gene expression, differential gene analysis and functional annotation; the detailed steps are listed in Supplementary file 3. MicroRNA sequencing mainly includes raw data processing, ncRNA and repetitive sequence annotation, reference genome comparison, miRNA identification, miRNA expression analysis, differential miRNA screening and target gene prediction; the detailed steps are shown in Supplementary file 4.

### Real-time fluorescence quantitative PCR analysis

Total RNA was extracted using the TRIzol (Invitrogen, USA) kit. The RNA integrity was detected by 1% agarose gel electrophoresis, RNA concentration and OD value were detected by Nanodrop 2000 Nucleic Acid Protein Analyzer (Thermo, USA). The qualified RNAs were stored at -80 °C. The PrimeScript RT Kit (Takara, Japan) was used for reverse transcription of gene, the Mir-X miRNA First-Strand Synthesis Kit (Takara, Japan) was used for miRNA reverse transcription, qRT-PCR was performed using SYBR Premix Ex Taq II Reagent (Takara) on a CFX96 Touch Detection System (Bio-Rad, CA, USA) [48, 49]. 2^−△△CT^ method was used to normalize the differentially expressed mRNAs and miRNAs [50], and finally Prism 6.0 software was used for graphing. All primers were designed using Primer Premier 5.0, and the primer sequence information is shown in Table 2.

**Table 2:** Sequence information of each primer

Name	Sequence (5’—3’)	Tm	Amplicon length
*β-actin*-F	GCCCCACCTGAGCGTAAATA	60.5	165
*β-actin*-R	GAGTCGGCGTGAAGTGGTAA		
*pdia*4-F	CAAATGATGTGCCTCACGAAA	57.5	144
*pdia*4-R	CATGCTCCTCCATAAATTTGCTA		
*canx*-F	TATGTCCTCACCGTGGCTCT	56.2	119
*canx*-R	TCCTCCTTCACATCCGGCTGA		
*dnajb*11-F	CAGTAGCCAAAGAAGCACCA	55.6	117
*dnajb*11-R	GGCACTCATCACAGACAAC		
*creld*2-F	CGTGTAAAGAGGATCAGTATTGC	57.0	163
*creld*2-R	CACTCATTCATATCGGTGCAT		
*calr*-F	CGCTTTGACGACTTCAGCAACA	61.2	176
*calr*-R	CCACAGATGTCAGGTCCGAA		
*calr*3*b*-F	GTCCACCCTGAAATCGCCAA	60.5	187
*calr*3*b*-R	CCTTTGTGACTCCCCATGTCT		
*hspa*5-F	ACCAAGCTGATCCCCAGGAA	60.2	273
*hspa*5-R	TCATGGCACGTTCACCTTCAA		
*hsp*90*b*1-F	GTGCTCTTCTTGCCTTCACATC	59.9	167
*hsp*90*b*1-R	AATCTGCGCTGCGTTCAATC		
*dnaja*-F	GGTTCTCCTCACCCATGGAC	59.8	192
*dnaja*-R	TCTTGCCACCATAACCATCACA		
*dnaja*1-F	GTGAAAGATGTGAAGGGCGTG	60.2	172
*dnaja*1-R	TCTCGGTGACTCATGCGTTG		
U6-F	GGAACGATACAGAGAAGATTAGC	58.5	94
U6-R	TGGAACGCTTCACGAATTTGCG		
oha-miR-199a-5p	GCCCAGTGTTCGGACTAC	56.4	84
aca-miR-199b-5p	GCCCAGTGTTCGGACTAC	56.5	82
novel-m1969-3p	TGGACGTCTCTGCTGAG	55.5	83
novel-m1546-3p	GGGAGTGGGGTGTTTG	56.4	83
cgr-miR-222-3p	CTACATCTGGCTACTGGGT	57.5	86
novel-m0481-5p	CGGAGCGGAGTGGAG	56.5	85
dre-miR-125b-2-3p	GCGGGTTGGGTTCTC	58.8	82
ssa-miR-125a-3p	GACGGGTTGGGTTCTC	56.0	83
pma-miR-20a-5p	GCAAAGTGCTTATAGTGCAG	55.4	84
xtr-miR-106	GCAGAAAAGTGCTTATAGTGCAG	57.0	85
ssa-miR-92b-5p	GAGGTGTGGGATGTGGT	55.6	85
dre-miR-15a-3p	CAGCAGGCCGTACTGT	55.5	83
novel-m1018-5p	GATGACAGTGTGGGTCCT	57.2	83

### Data analysis

Data were processed and evaluated using SPSS 22.0 software with one-way analysis of variance (ANOVA) and independent t-test (*P* < 0.05 = significance). All data were expressed as mean ± standard deviation (SD).

### Electronic supplementary material

Below is the link to the electronic supplementary material.

Supplementary Material 1

Supplementary Material 2

Supplementary Material 3

Supplementary Material 4

Supplementary Material 5

## Data Availability

The original contributions presented in the study are publicly available. All raw sequencing data have been deposited in the NCBI Sequence Read Archive (SRA) under accession numbers SRR28285189 to SRR28285197 for transcriptomics, and SRR28262212 to SRR28262220 for miRNA sequencing.

## Abbreviations

NCBI: National Center for Biotechnology Information
qRT-PCR: Real-time quantitative reverse transcription polymerase chain reaction
GO: Gene ontology
KEGG: Kyoto Encyclopedia of Genes and Genomes
Wnt: Wingless and INT-1
