# Supplementary material for: Integrated transcriptome and microRNA analysis reveals molecular responses to high-temperature stress in the liver of American shad (Alosa sapidissima)
Source: BMC Genomics. 2024 Jul 1;25:656. doi: 10.1186/s12864-024-10567-w (PMC11218383; doi:10.1186/s12864-024-10567-w)
Supplement: Supplementary file 3 — Supplementary Material 3 [file 12864_2024_10567_MOESM3_ESM.docx]

**Introduction to the methodology and steps of participant transcriptome analysis**

**I. Analysis steps and parameterization**

**1. Data quality control:**

QC: Raw data were OC'd using fastp (v0.20.0) with the following parameters: -q 5 -n 5

(1) Number of unknown bases N<5;

(2) Remove sequences with base mass values less than 5 for 50% of the length of the Reads;

(3) Remove the connector sequence.

**2. Reference genome selection**

(1) Reference genome: American shad (*Alosa sapidissima*);

(2) Download existing reference genomes from relevant websites (NCBl, JGl, Ensemble, websites of individual sections, etc.). Generally, download the reference genomes according to the frequency of use of the genomes, choosing the more commonly used and the latest version of the reference genome.

**3. Comparison of reference genomes**

(1) The reference genome was compared using HISAT2 (v2.1.0), and the parameter setting was: --novel-splicesite-outfile XX.ss. The sam file was converted to bam format using the software samtools (version 1.9). The rnaseq subroutine of qualimap (v2.2.1) was then used to count the results and determine the efficiency of the comparison.

**4. Transcript assembly**

(1) Use StringTie (stringtie-2.1.3b.Linux_x86_64) software to assemble transcripts based on bam files, set parameters: -f 0.2 -a 30 -c 5 -j 5;

(2) Use StringTie to fuse the reconstruction results of all samples to get the structure-optimized transcript structure annotation file, set the parameter --merge.

**5. Quantification of gene expression**

(1) Use StringTie to quantify gene expression based on the annotation file optimized for gene structure and the bam file obtained by comparison, set parameters: -e -p 4;

(2) The above results were collated using Ballgown (R script) to get the gene expression of the readcount matrix;

(3) Use your own script to get the fpkm matrix.

**6. Analysis of variance**

Differential analyses were performed using the readcount matrix and were categorized as having biological duplicates and not having biological duplicates.

(1) Biological duplicates: Differences were analyzed using DESeq2 software (R script);

(2) Without biological duplicates: Differences were analyzed using edgeR software (R script) with the parameter --dispersion 0.05;

(3) Differential gene screening: screening criteria: |Log2FC|>=1 and fdr<=0.05; use R (v3.6.2) ggplot2 program package to draw MA plots, volcano plots, box plots, and gene expression clustering heatmaps using R (v3.6.2) pheatmap.

**7. Functional annotation of differential genes**

(1) Annotate the GO, KEGG, COG, KOG, NR, Swissprot, Pfam databases with BLASTALL (v2.2.26, with value set to 1e-5) to analyze the function of differential genes, and then used our own Perl script to count the number of differential genes annotated to each database and make a graph.

(2) Enrichment analysis of differential genes annotated to KEGG databases was performed using our own scripts, and KEGG enrichment scatter plots of the top 20 most significant pathways were made using R (v3.6.2). KEGG secondary pathways on annotations were mapped using our own SVG script;

(3) Enrichment analysis of differential genes annotated to the GO database using our own scripts, enrichment analysis of BP, CC, and MF using fisher's exact test, and mapping of the number of GO annotated functions of genes using Perl's SVG module.

**8. Variable shear analysis**

(1) With biological duplicates: Variable shear analysis was performed using rMATs (rMATS.4.0.2) software based on the bam file and gene structure annotation file with the following parameters: -t paired --readLength 150;

(2) Biology-free duplication: Variable shear analysis was performed using astalavista software (astalavista-4.0) based on the optimized gene structure files.

**9. SNP/INDEL Detection**

(1) Use bcftools, the software that comed with samtools (v1.9), to analyze SNP and INDEL. First, used bcftools mpileup to merge the bam files to generate bcf files, parameters: bcftools mpileup --annotate AD, DP --adjust-MQ 50 --redo-BAQ --min-BQ 13 --min-MQ 1 --output-type v . Then used bcftools to generate a bcf file into the common vcf format with the following parameters: bcftools call ---multiallelic-caller --variants-only --output-type v.

**II. References:**

[1] Deng YY, Li JQ, Wu SF, Zhu YP, et al. Integrated nr Database in Protein Annotation System and Its Localization. Computer Engineering. 2006, 32(5):71-74.

[2] Apweiler R, Bairoch A, Wu CH, et al. UniProt: the universal protein knowledgebase. Nucleic acids research. 2004, 32: D115-D119.

[3] Finn RD, Bateman A, Clements J, et al. Pfam: the protein families database. Nucleic acids research. 2013: gkt1223.

[4] Tatusov RL, Galperin MY, Natale D A. The COG database: a tool for genome scale analysis of protein functions and evolution. Nucleic Acids Research. 2000, 28(1):33-36.

[5] Koonin EV, Fedorova ND, Jackson JD, et al. A comprehensive evolutionary classification of proteins encoded in complete eukaryotic genomes. Genome biology. 2004, 5(2): R7.

[6] Ashburner M, Ball C A, Blake J A, et al. Gene ontology: tool for the unification of biology. Nature genetics. 2000, 25(1): 25-29.

[7] Kim Daehwan,Paggi Joseph M,Park Chanhee et al. Graph-based genome alignment and genotyping with HISAT2 and HISAT-genotype.[J] .Nat. Biotechnol., 2019, 37: 907-915.

[8] Pertea Mihaela,Kim Daehwan,Pertea Geo M et al. Transcript-level expression analysis of RNA-seq experiments with HISAT, StringTie and Ballgown.[J] .Nat Protoc, 2016, 11: 1650-67. [9] Zhou Katherine I,Shi Hailing,Lyu Ruitu et al. Regulation of Co-transcriptional Pre-mRNA Splicing by mA through the Low-Complexity Protein hnRNPG.[J] .Mol. Cell, 2019, undefined: undefined.

[10] Robinson, M. D. McCarthy, D. J. Smyth, G. K, et al. edgeR: a Bioconductor package for differential expression analysis of digital gene expression dataBioinformatics. Anders S., and Huber W. (2010). Differential expression analysis for sequence count data. Genome Biol.
