# Supplementary material for: Integrated transcriptome and microRNA analysis reveals molecular responses to high-temperature stress in the liver of American shad (Alosa sapidissima)
Source: BMC Genomics. 2024 Jul 1;25:656. doi: 10.1186/s12864-024-10567-w (PMC11218383; doi:10.1186/s12864-024-10567-w)
Supplement: Supplementary file 4 — Supplementary Material 4 [file 12864_2024_10567_MOESM4_ESM.docx]

**Introduction to sRNA analysis methods and procedures**

**I.Analysis steps and parameter settings**

**1. the original data preprocessing**

Use the software Cutadapt (v1.18) to filter the raw Reads (Raw Data), the filtering criteria:

(1) Intercept the sequencing connector (adapter) in Reads;

(2) Filter low-quality data to ensure that the data quality removes N (N indicates that base information cannot be determined) from a percentage of Reads greater than 10%;

(3) Remove Reads with an end quality value of Q<20;

(4) Animals to remove sequences shorter than 15 or longer than 35 bases of the sequence.

The high quality Reads obtained after the above series of filtration were called Clean Data.

**2. ncRNA and repetitive sequence annotation**

Bowtie (v1.2.2) was used to compare Clean Data with miRBase (v21), Rfam (v14.1) and Repbase databases, and identify ncRNAs and repeat sequences, such as miRNA (conserved miRNA), ribosomal RNA (rRNA), transfer RNA (tRNA), small nuclear RNAs (snRNA), and small nucleolar RNAs (snoRNA), and the order of identification was: miRNA>rRNA>tRNA>snRNA>snoRNA>repeat. Parameter settings: -v 2 -al *.mapped.seq.txt -un *.unmapped.seq.txt.

**3. Comparison with reference genome**

Use the software Bowtie (v1.2.2) to compare the unannotated sequences of ncRNA database in 2 that meet the requirement of depth>2 with the reference genome sequences, with the parameter setting: -a --best -strata.

Use the software Bedtools (v2.17.0) to obtain sequences from the reference genome without annotation information. Parameter setting: bedtools intersect -a mapped_genome -b gff_file -wa -wb .

**4. miRNA identification**

The miRNA identification was divided into two aspects:

(1) Conservative miRNA identification, i.e., in 2, the conserved miRNAs were obtained by comparing the miRNA sequences in the miRBase library;

(2) Identification of non-conserved miRNAs, the sequences obtained in 3 were used to predict new miRNAs using the software Mireap (v0.2) [4], with the following parameter settings: default parameters.

**5. miRNA base preference analysis**

The first base at the 5' end of the sequence was statistically analyzed using a self-developed perl program to analyze base preference.

**6. miRNA base editing analysis**

A self-developed perl program was used to look for differences between the miRNA sequences of each sample and the corresponding conserved miRNA mature bodies and their precursor sequences, and to find miRNAs that may undergo base editing.

**7. miRNA family analysis**

Using the self-developed perl program, we performed miRNA family analysis on the identified miRNAs and counted the number of each miRNA family.

**8. miRNA target gene prediction**

In animals, Miranda (v3.3a) software was used to predict the target genes of miRNAs, with parameters set to: -sc 140 -en -30.

**9. miRNA expression analysis**

The number of miRNA counts of the samples was counted using a self-developed perl program, and the expression was normalized using the TPM algorithm.
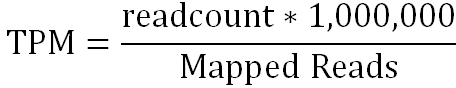


The processing formula for TPM was as follows: in the formula, readcount denoted the number of reads compared to a particular miRNA; Mapped Reads denoted the total number of miRNA reads compared to the total number of miRNA reads on the sample.

(1) The density distribution of TPM was plotted using the plt.plot function in the plotting library Matplotlib in python (v2.7.18);

(2) The cor function in R (v3.6.2) was used to calculate the correlation coefficient r by doing the pearson correlation test on the TPM matrix of each sample, and the correlation coefficients were plotted as heat maps using a self-developed perl program;

(3) The princomp function in R (v3.6.2) was used to perform principal component analysis, i.e., dimensionality reduction, and the principal component analysis was plotted using plot.

**10. Differential miRNA screening**

For data with biologically replicated samples, differential fold change (FC, Fold Change) was calculated using the DESeq2 package in R (v3.6.2); biology-free duplicate samples were used to calculate multiplicity of differences using the edgeR program package in R (v3.6.2). Threshold setting: filtered out genes for which the sum of the readcount values of the compared two groups was less than 10.

Filtering criteria: |log2(FC)| ≥ 1 and FDR (obtained by Benjamini-Hochberg correction for significance of difference p-value) < 0.05.

(1) MA plots and volcano plots were drawn using the R (v3.6.2) ggplot2 program package;

(2) Heatmap of differential miRNA clustering using self-developed python program. Clustering method: hierarchical cluster.

**11. Differential miRNA target gene annotation**

(1) After screening the differential miRNAs, find the target genes corresponding to the differential miRNAs;

(2) Analyze the GO enrichment of differential miRNA targeted genes according to the principle of hypergeometric distribution, and used the self-developed perl program to draw differential miRNA target gene annotations;

(3) Differential miRNA target genes were analyzed for topGO enrichment using the R (v3.6.2) topGO package, and directed acyclic maps were plotted;

(4) KEGG enrichment analysis of differential miRNA target genes was performed according to the principle of hypergeometric distribution and plot using the R (v3.6.2) ggplot2 program package to plot the KEGG annotation classification statistics and enrichment scatter plots of differential miRNA target genes, and the enrichment factor was calculated by the formula:(number of differential genes in the pathway/total number of genes annotated to the pathway)/(total number of differential genes/total number of genes).

**II. References:**

[1] Kechin A, Boyarskikh U, Kel A, Filipenko M. cutPrimers: A New Tool for Accurate Cutting of Primers from Reads of Targeted Next Generation Sequencing. J Comput Biol. 2017 Nov;24(11):1138-1143.

[2] Langmead B. Aligning short sequencing reads with Bowtie. Current protocols in bioinformatics, 2010, 11.7. 1-11.7. 14.

[3] Quinlan AR, Hall IM. BEDTools: a flexible suite of utilities for comparing genomic features. Bioinformatics. 2010 Mar 15;26(6):841-2.

[4] Thiel T, Michalek W, Varshney R, Graner A(2003) Exploiting EST databases for the development and characterization of gene-derived SSR-markers in barley (Hordeum vulgare L.). Theoretical and Applied Genetics 106 (3): 411-422. [4] Li Y, Wan L, Bi S, Wan X, Li Z, Cao J, Tong Z, Xu H, He F, Li X. Identification of Drought-Responsive MicroRNAs from Roots and Leaves of Alfalfa by High-Throughput Sequencing. Genes (Basel). 2017 Apr 13;8(4):119.

[5] Erickson SL, Salekin KL, Johnson LN, Doran SC. The predictive power of intelligence: Miranda abilities of individuals with intellectual disability. Law Hum Behav. 2020 Feb;44(1):60-70.

[6] Danecek P, McCarthy SA. BCFtools/csq: haplotype-aware variant consequences. Bioinformatics. 2017 Jul 1;33(13):2037-2039.
