# Supplementary material for: Integrated transcriptome and microRNA analysis reveals molecular responses to high-temperature stress in the liver of American shad (Alosa sapidissima)
Source: BMC Genomics. 2024 Jul 1;25:656. doi: 10.1186/s12864-024-10567-w (PMC11218383; doi:10.1186/s12864-024-10567-w)
Supplement: Supplementary file 5 — Supplementary Material 5 [file 12864_2024_10567_MOESM5_ESM.docx]

**transcriptome**


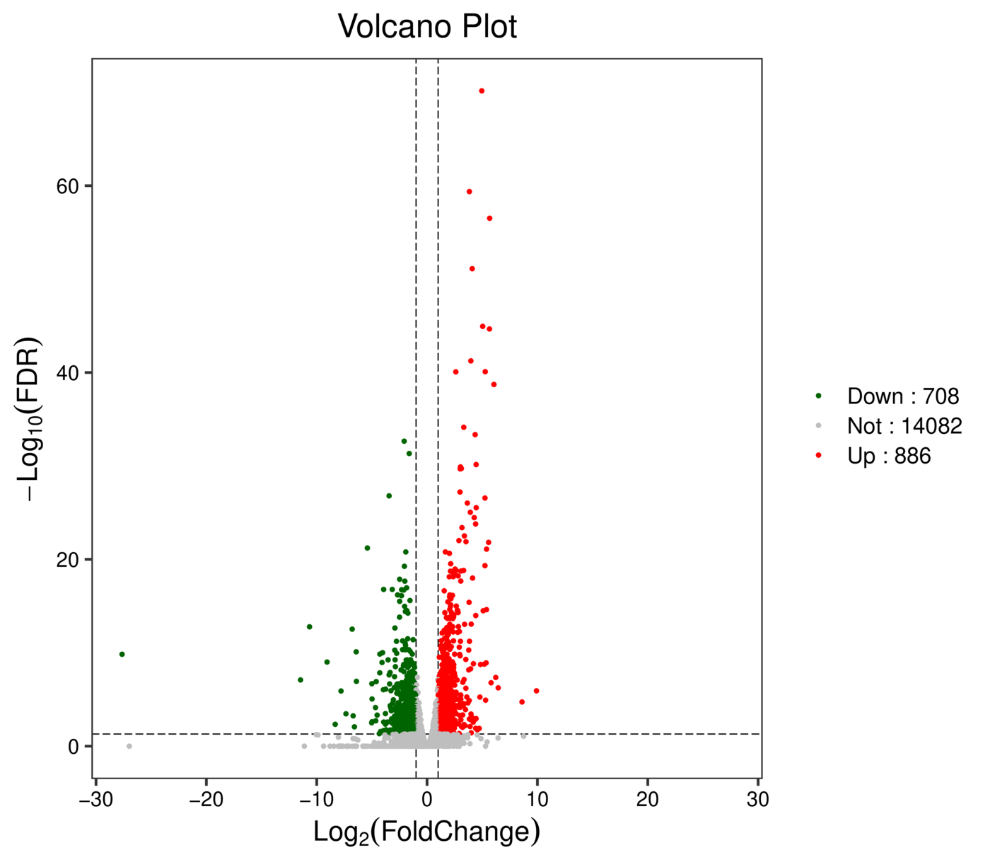


Differential expression volcano plot in LowLi vs. MidLi comparison group.


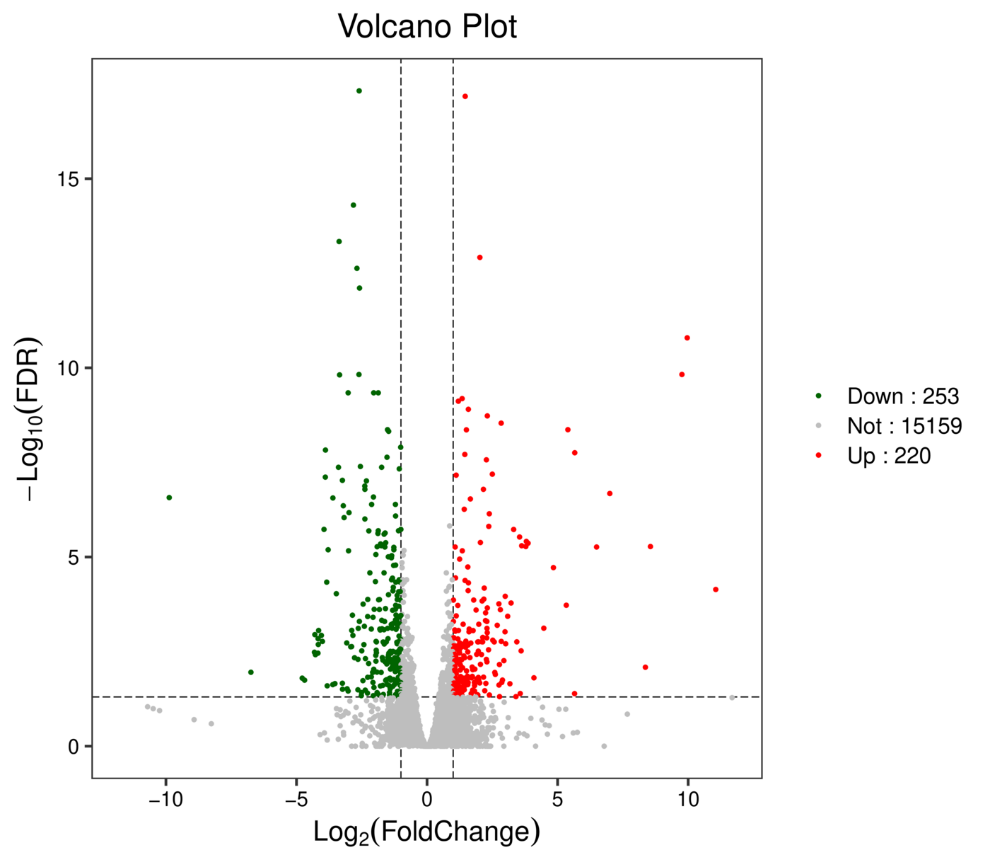


Differential expression volcano plot in MidLi vs. HighLi comparison group.


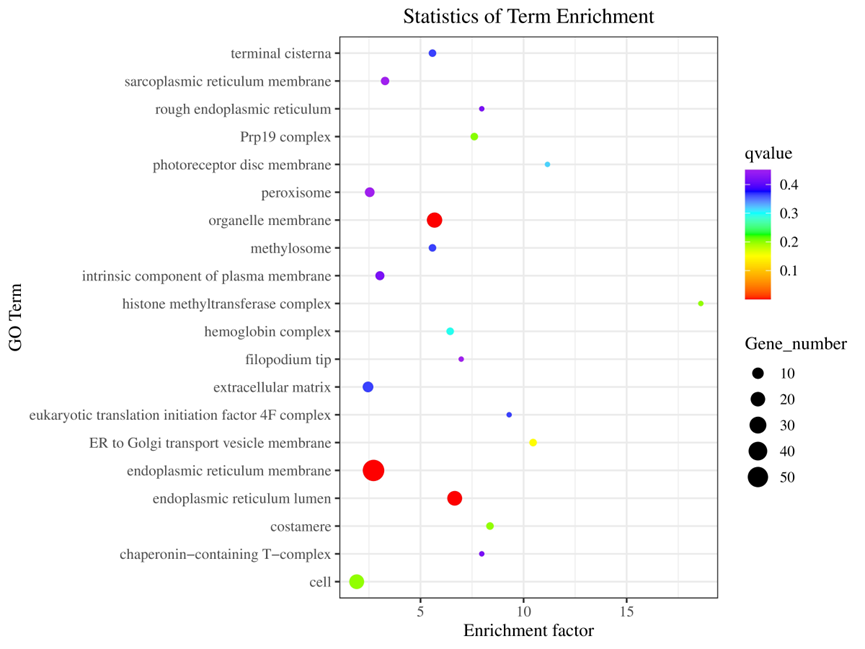


Scatter plot of GO pathway enrichment (cellular components) of DEGs in LowLi vs. HighLi group.


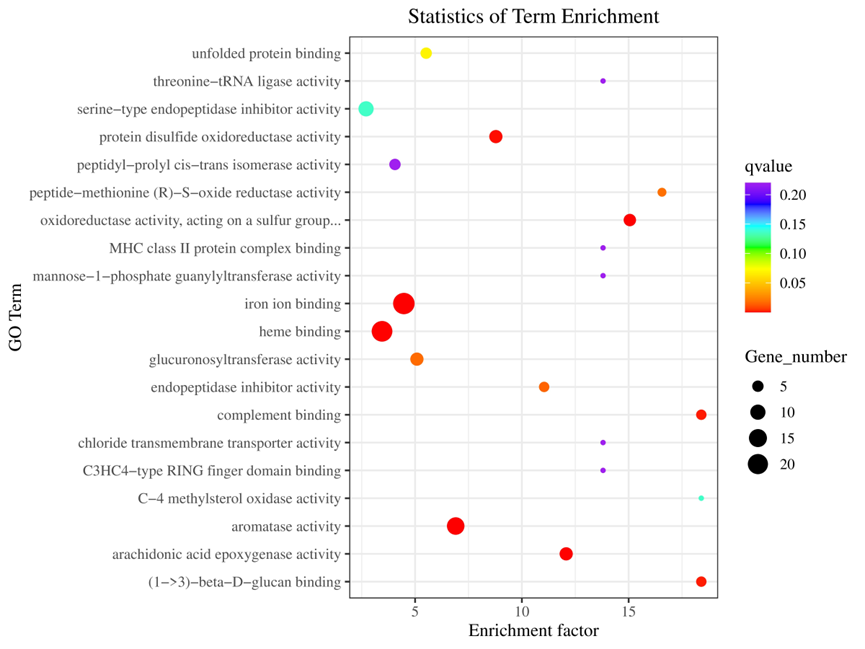


Scatter plot of GO pathway enrichment (molecular function) of DEGs in LowLi vs. HighLi group.


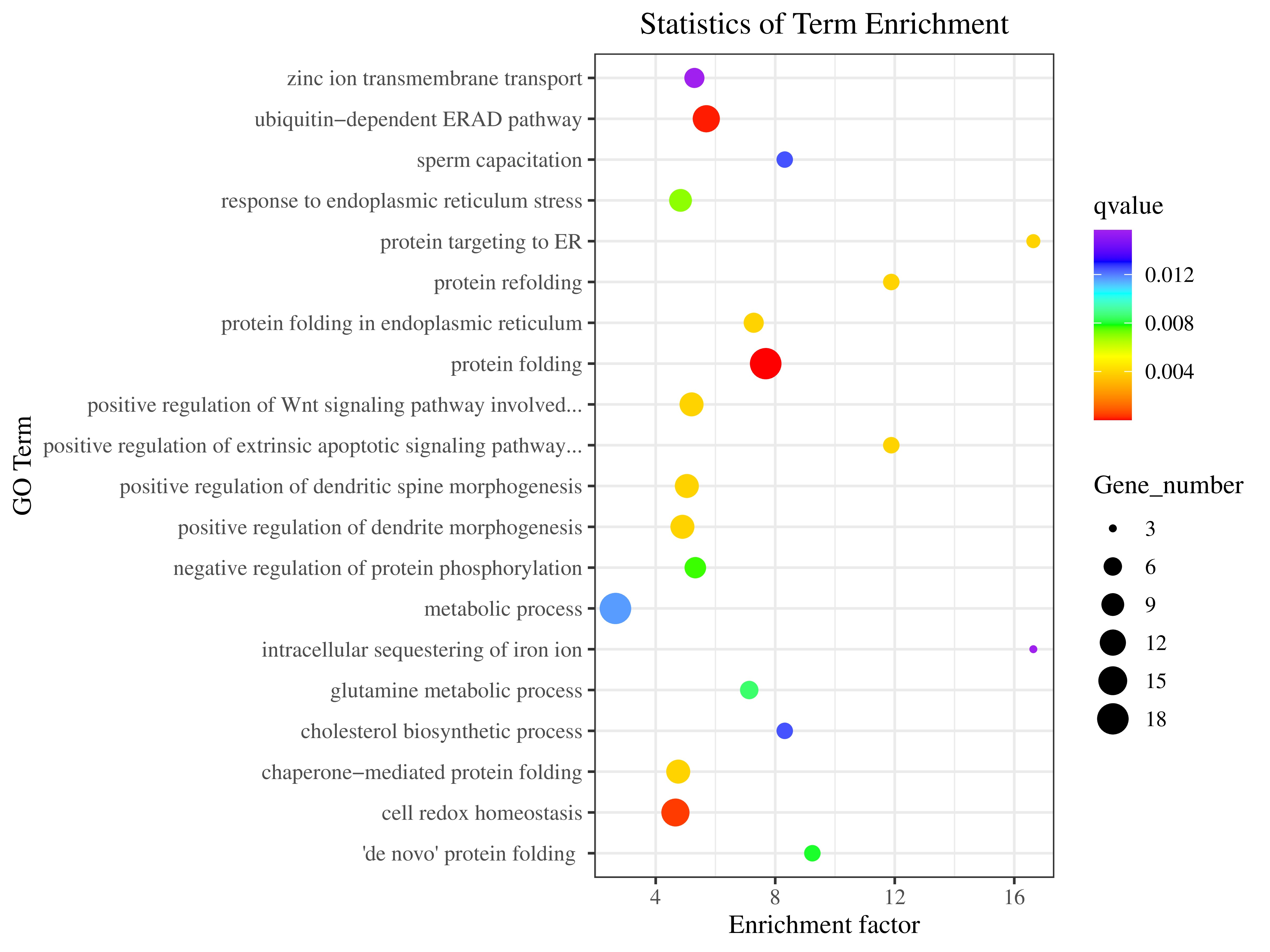


Scatter plot of GO pathway enrichment (biological processes) of DEGs in LowLi vs. MidLi group.


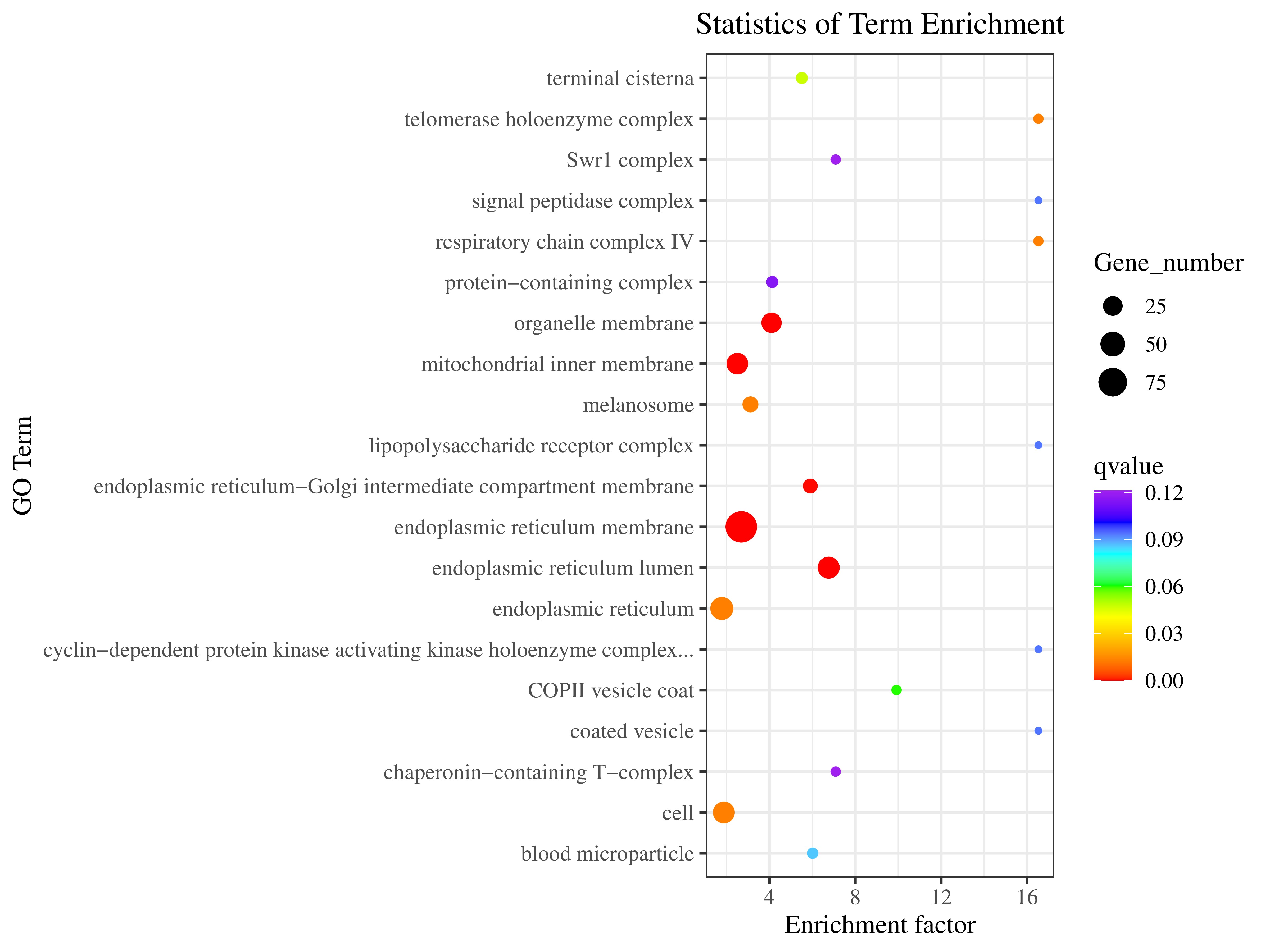


Scatter plot of GO pathway enrichment (cellular components) of DEGs in LowLi vs. MidLi group.


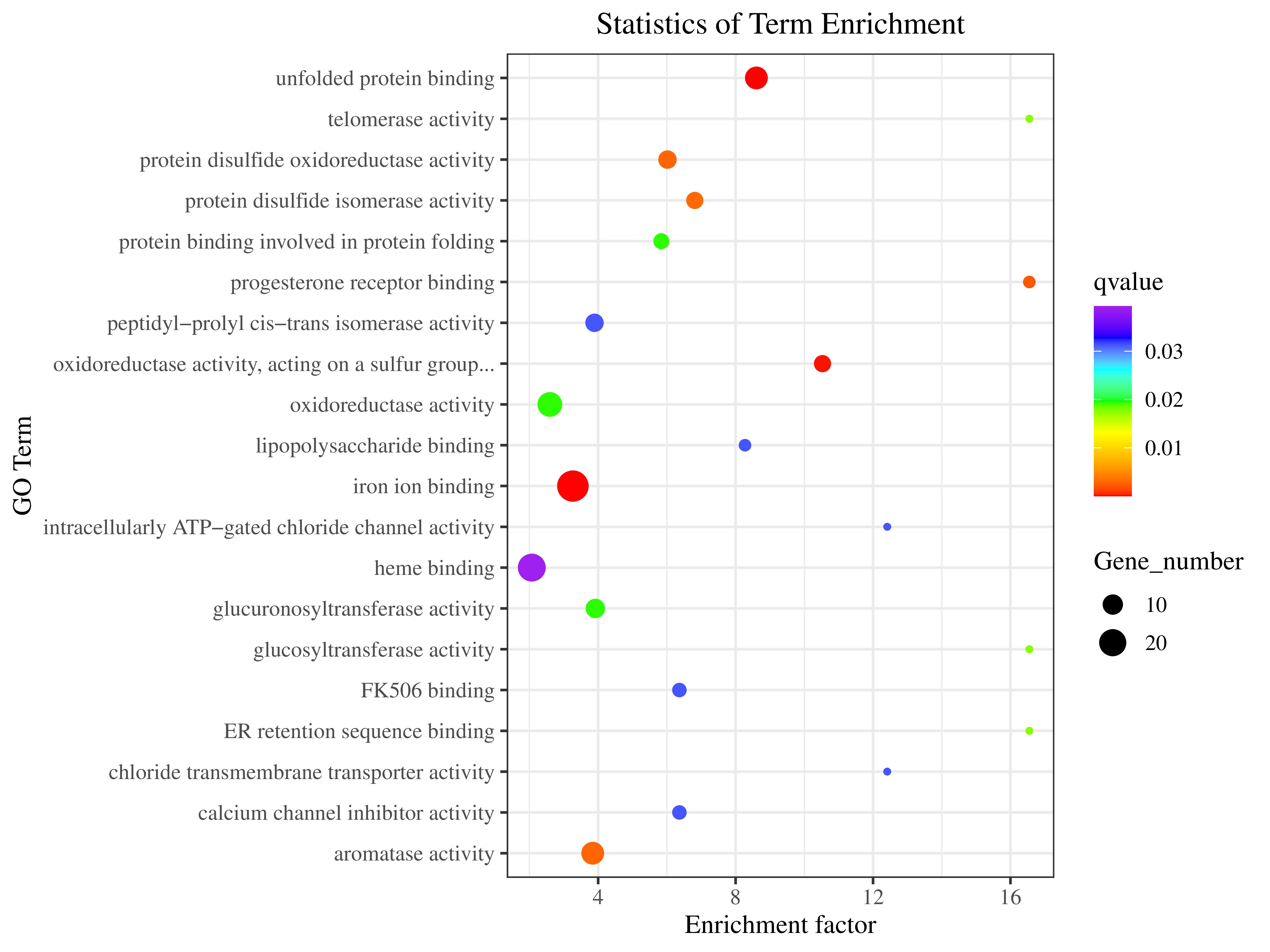


Scatter plot of GO pathway enrichment (molecular function) of DEGs in LowLi vs. MidLi group.


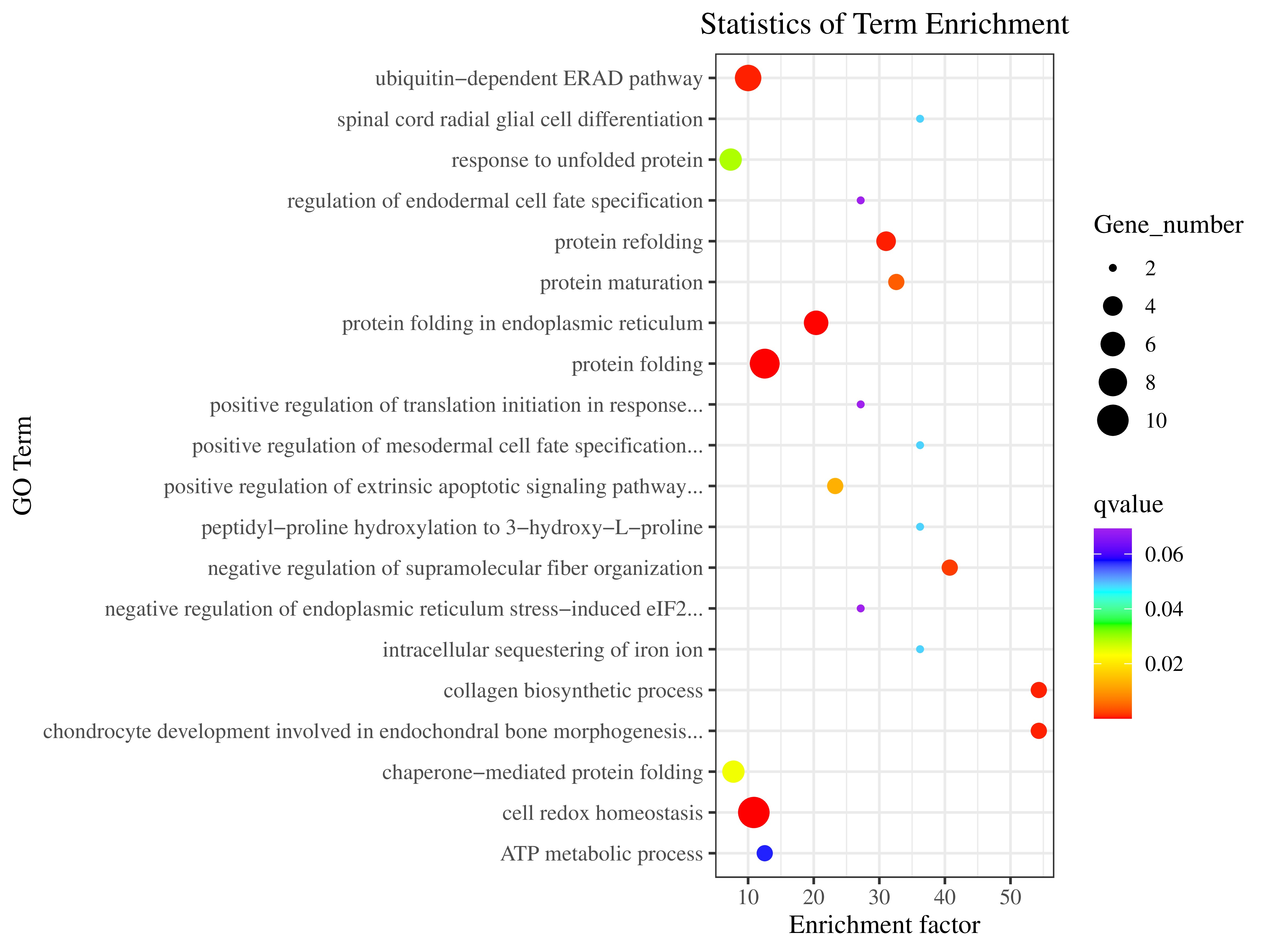


Scatter plot of GO pathway enrichment (biological processes) of DEGs in MidLi vs. HighLi group.


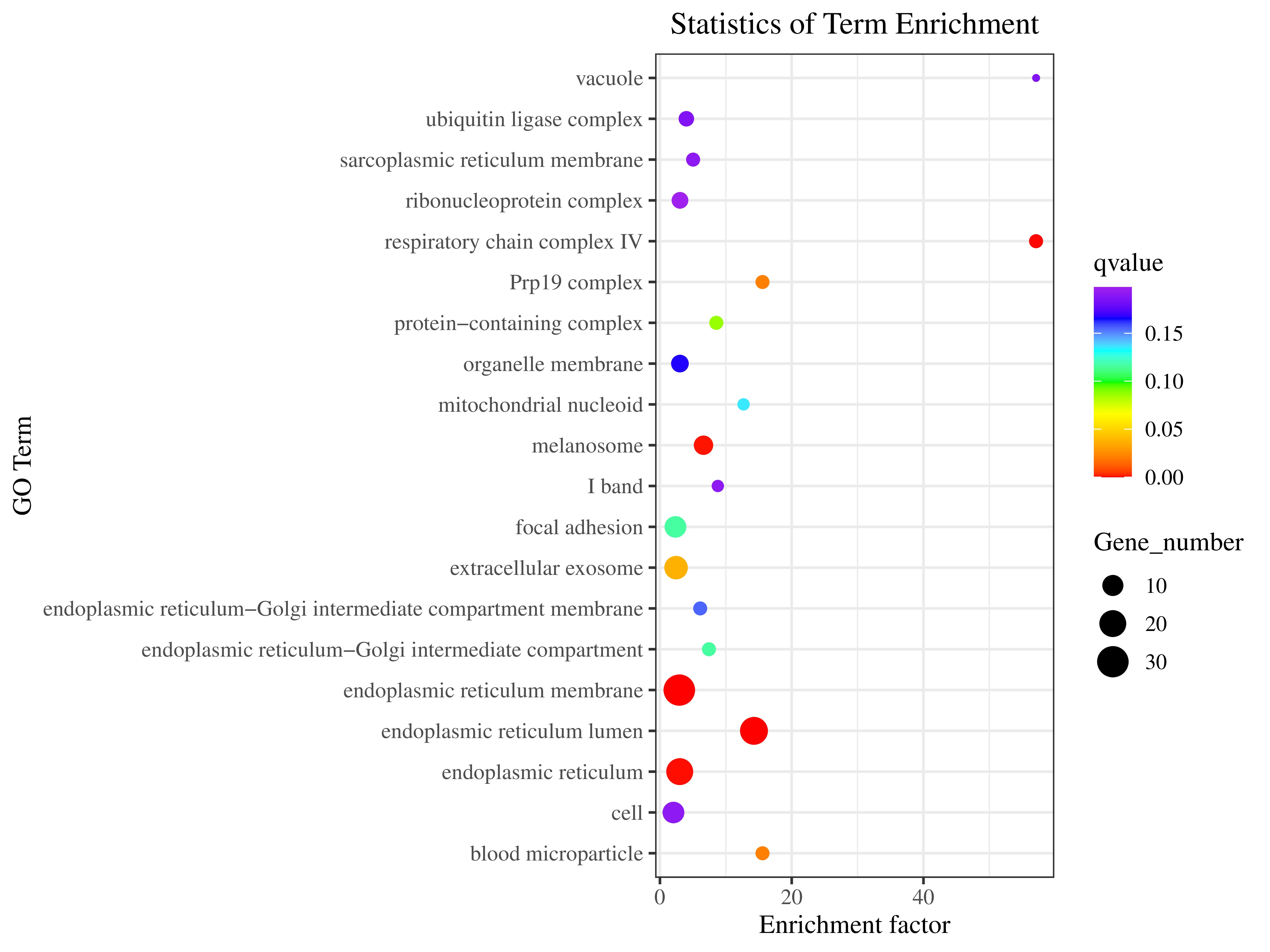


Scatter plot of GO pathway enrichment (cellular components) of DEGs in MidLi vs. HighLi group.


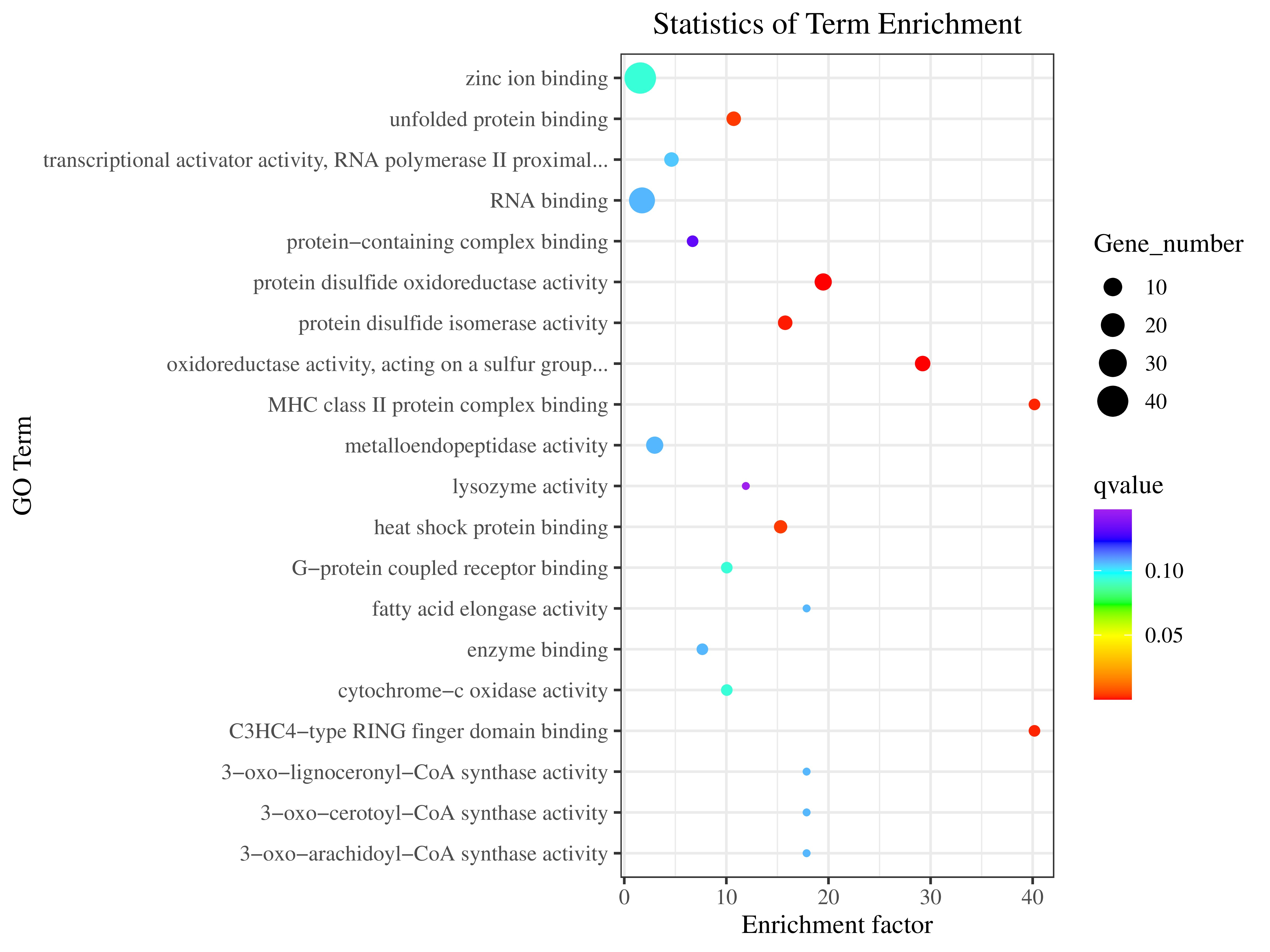


Scatter plot of GO pathway enrichment (molecular function) of DEGs in MidLi vs. HighLi group.


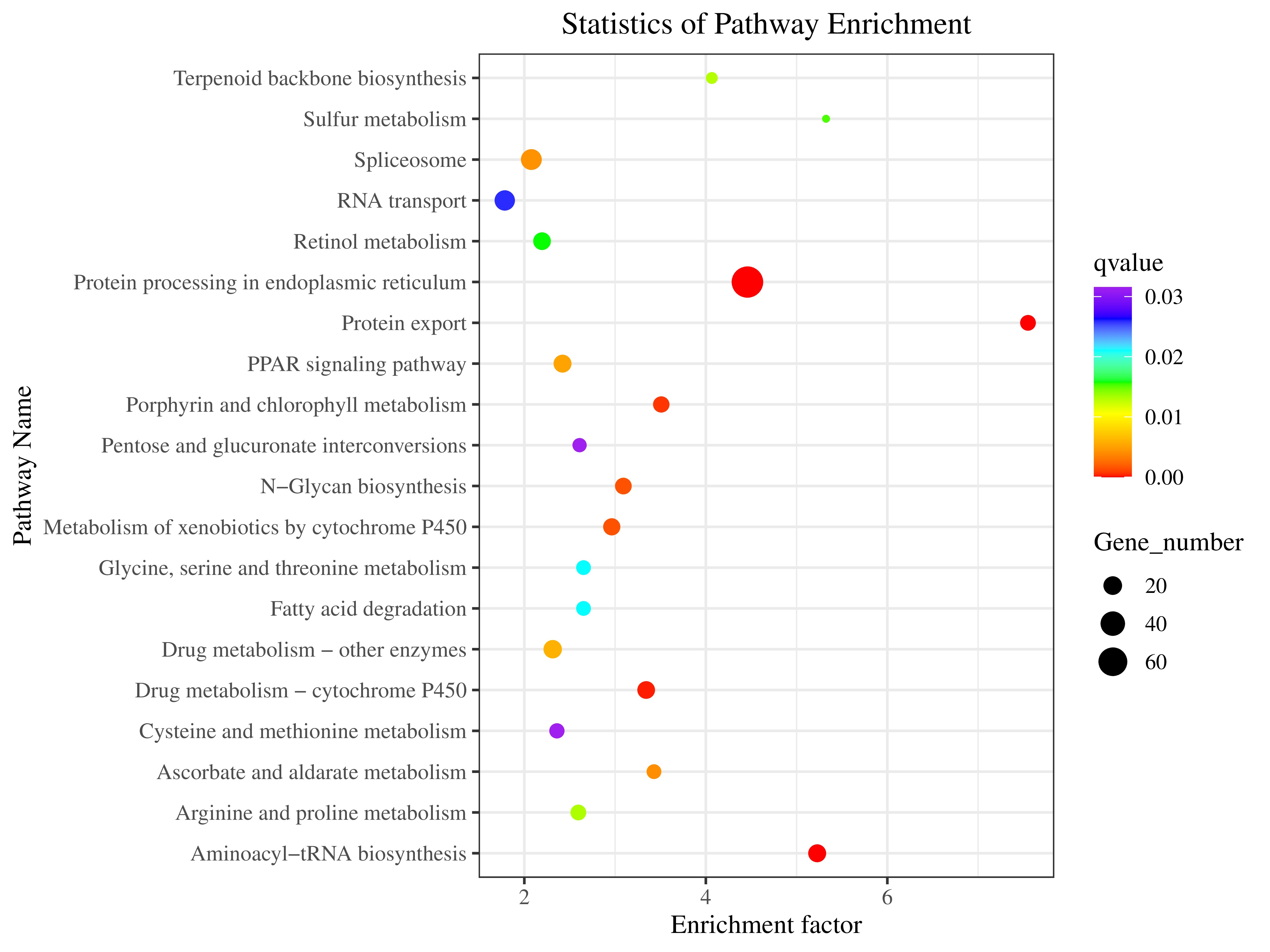


KEGG pathway enrichment scatter plot of differentially expressed genes in LowLi vs. MidLi group.


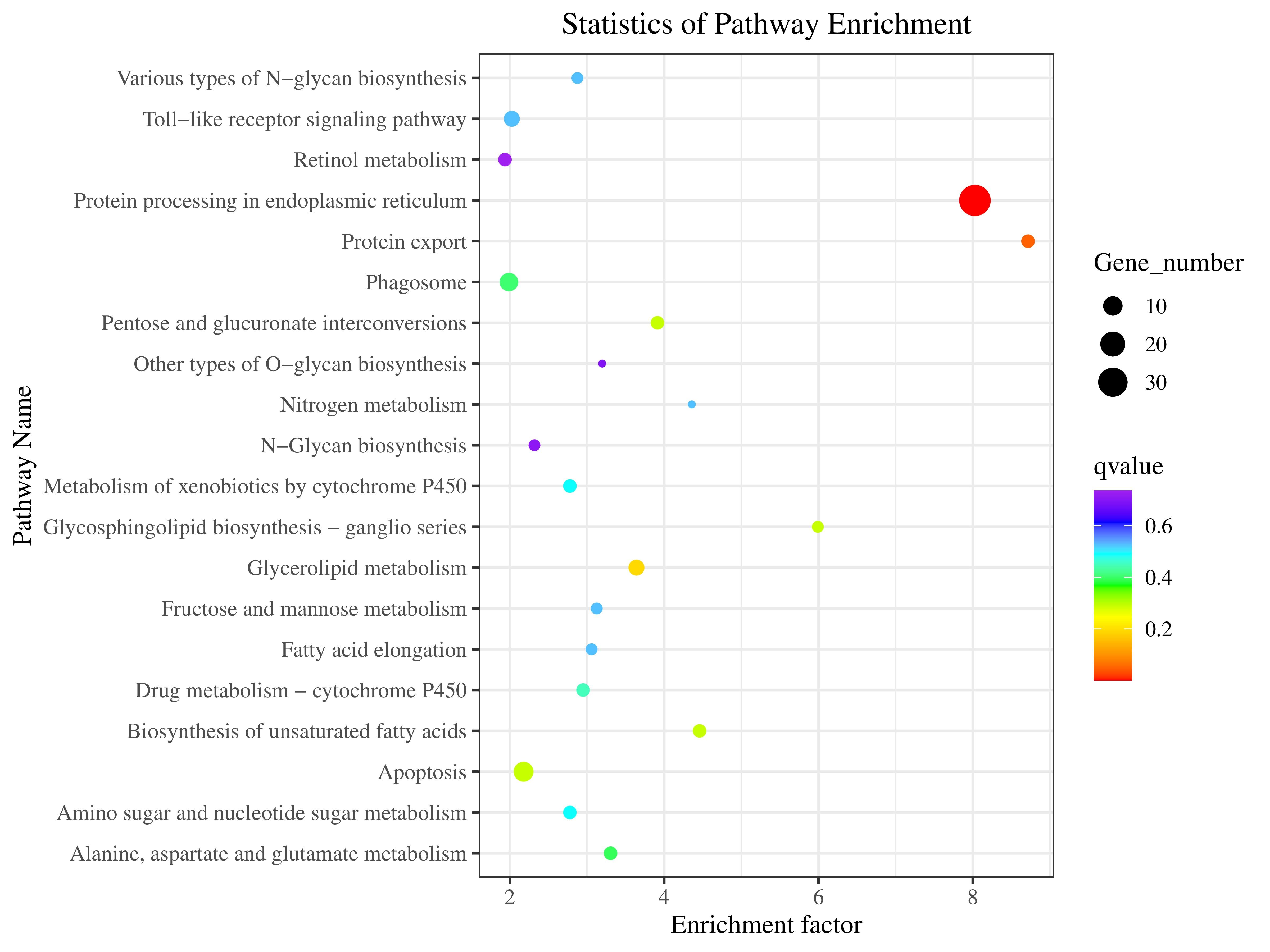


KEGG pathway enrichment scatter plot of differentially expressed genes in MidLi vs. HighLi group.


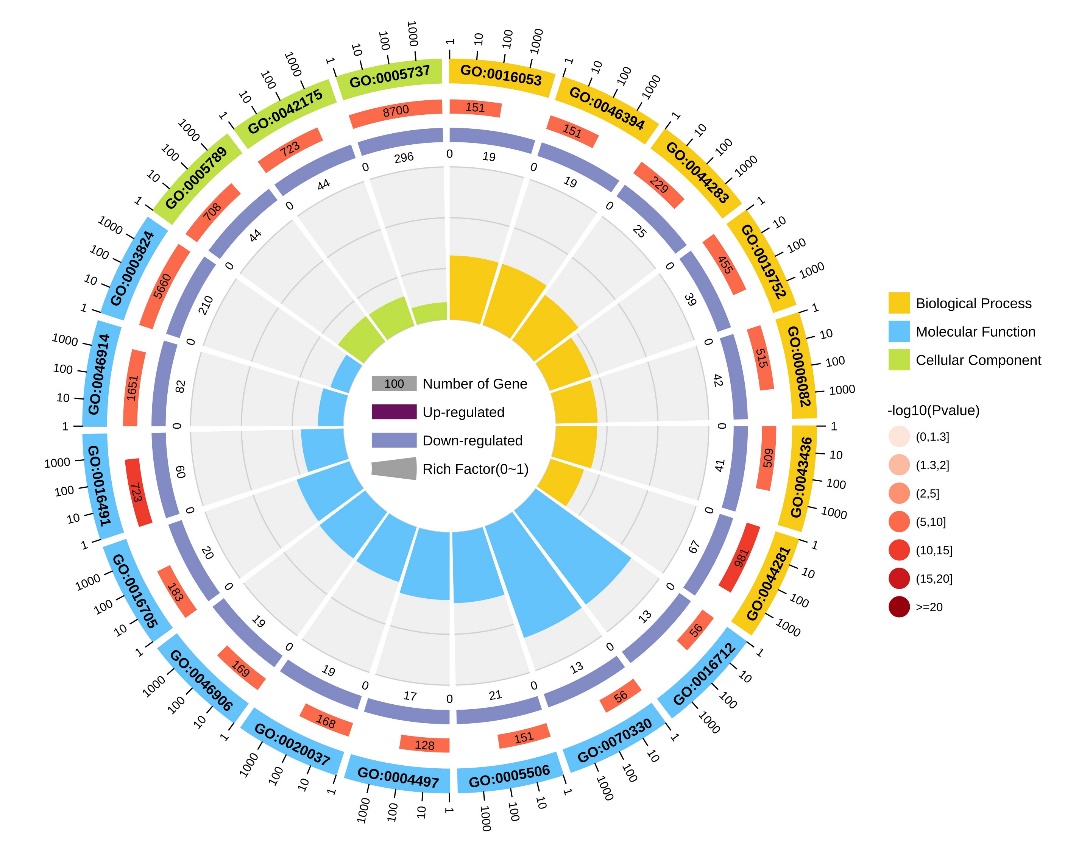


Circle diagram of enrichment of down-regulated genes GO in LowLi vs. MidLi group.


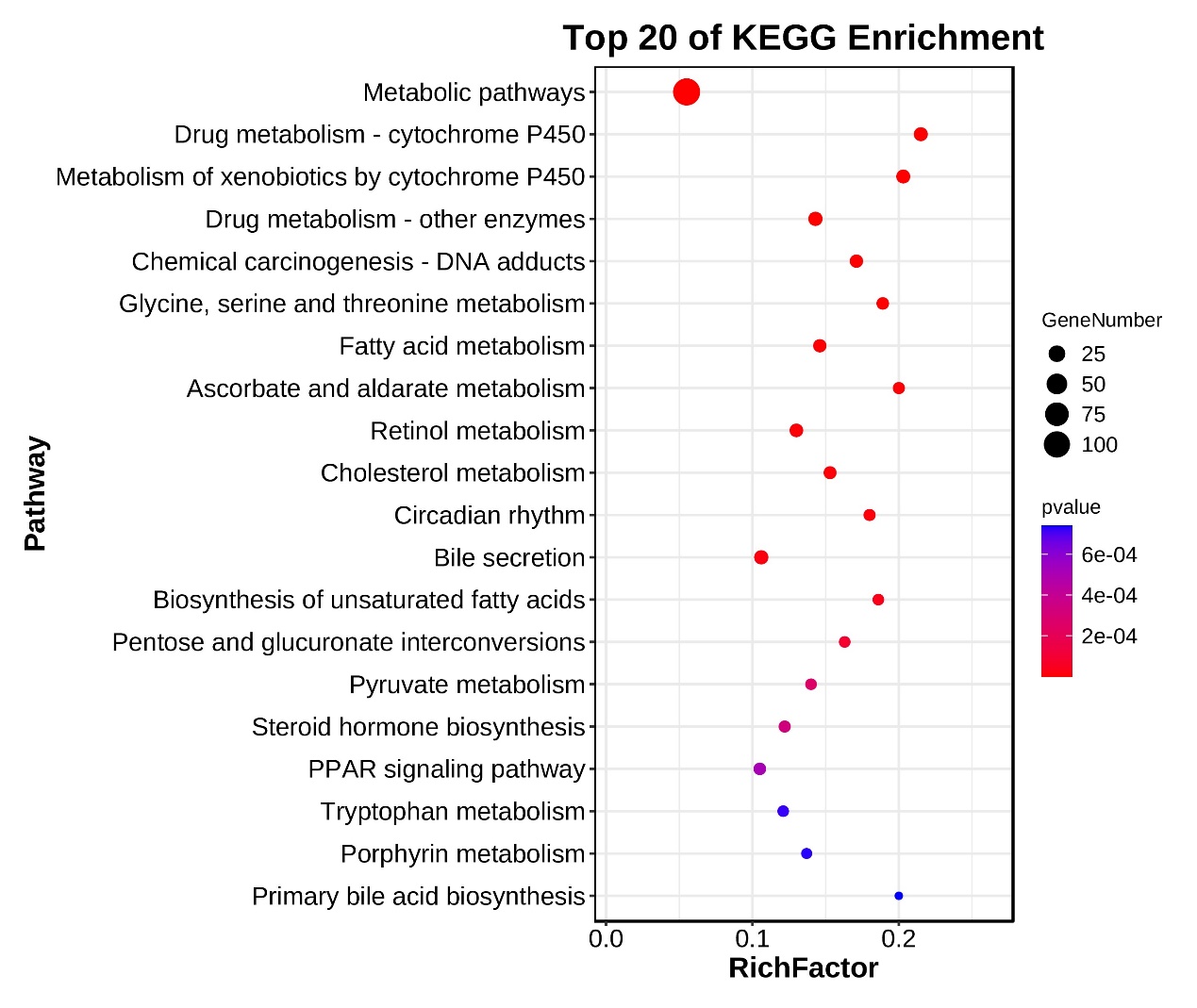


KEGG pathway enrichment scatter plot for LowLi vs. MidLi down-regulated genes.


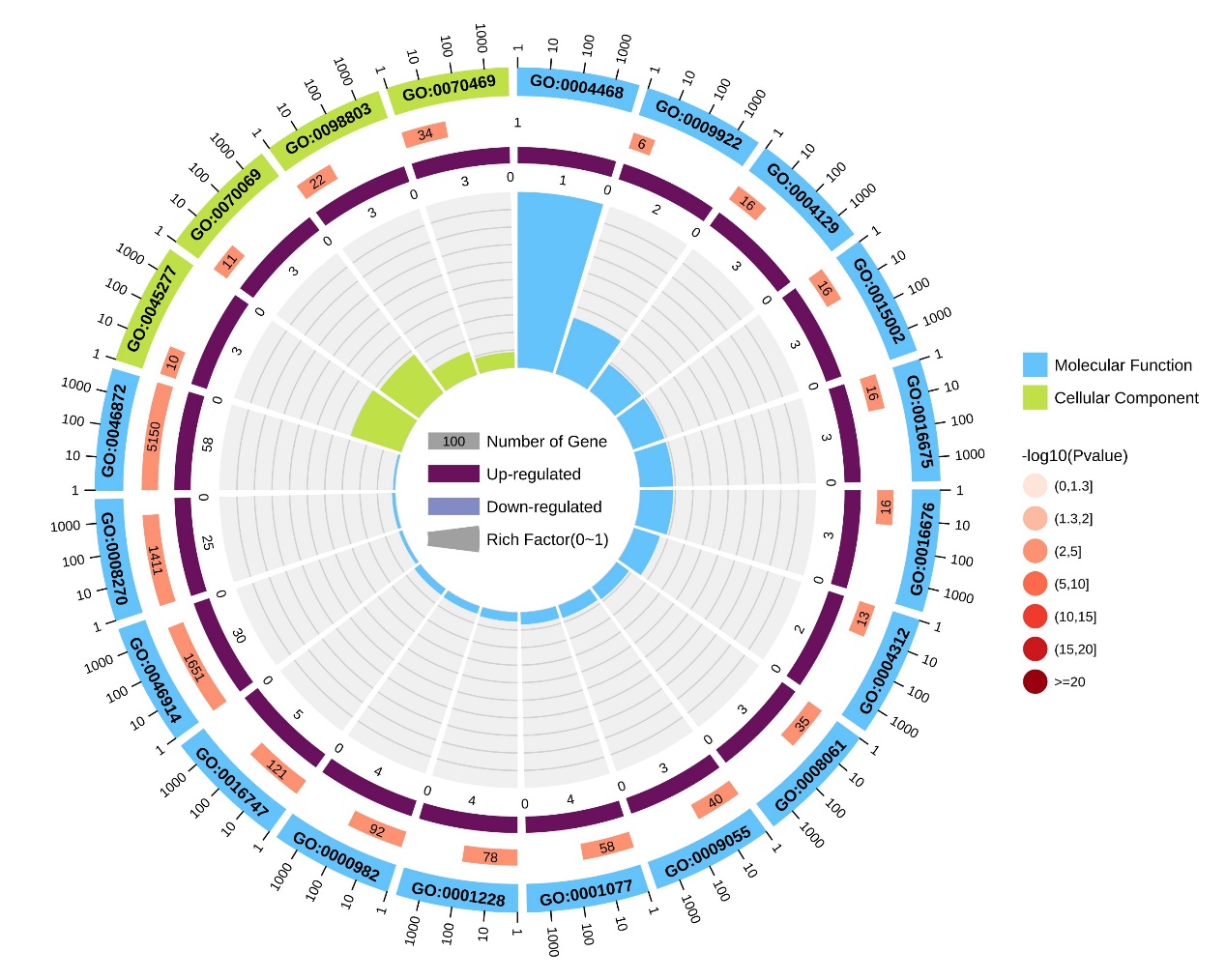


Circle diagram of enrichment of up-regulated genes GO in MidLi vs. HighLi.


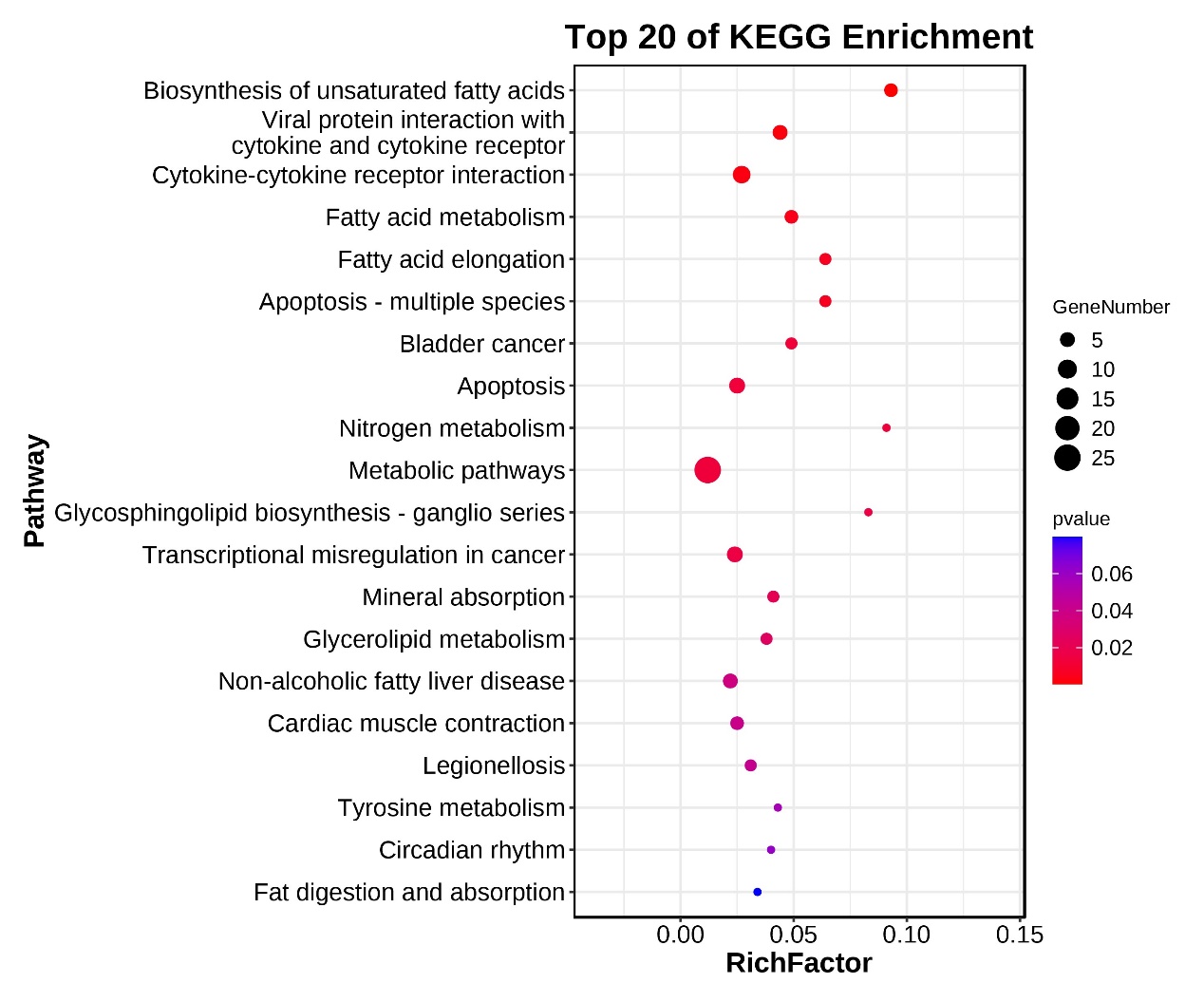


KEGG pathway enrichment scatter plot for MidLi vs. HighLi up-regulated genes.


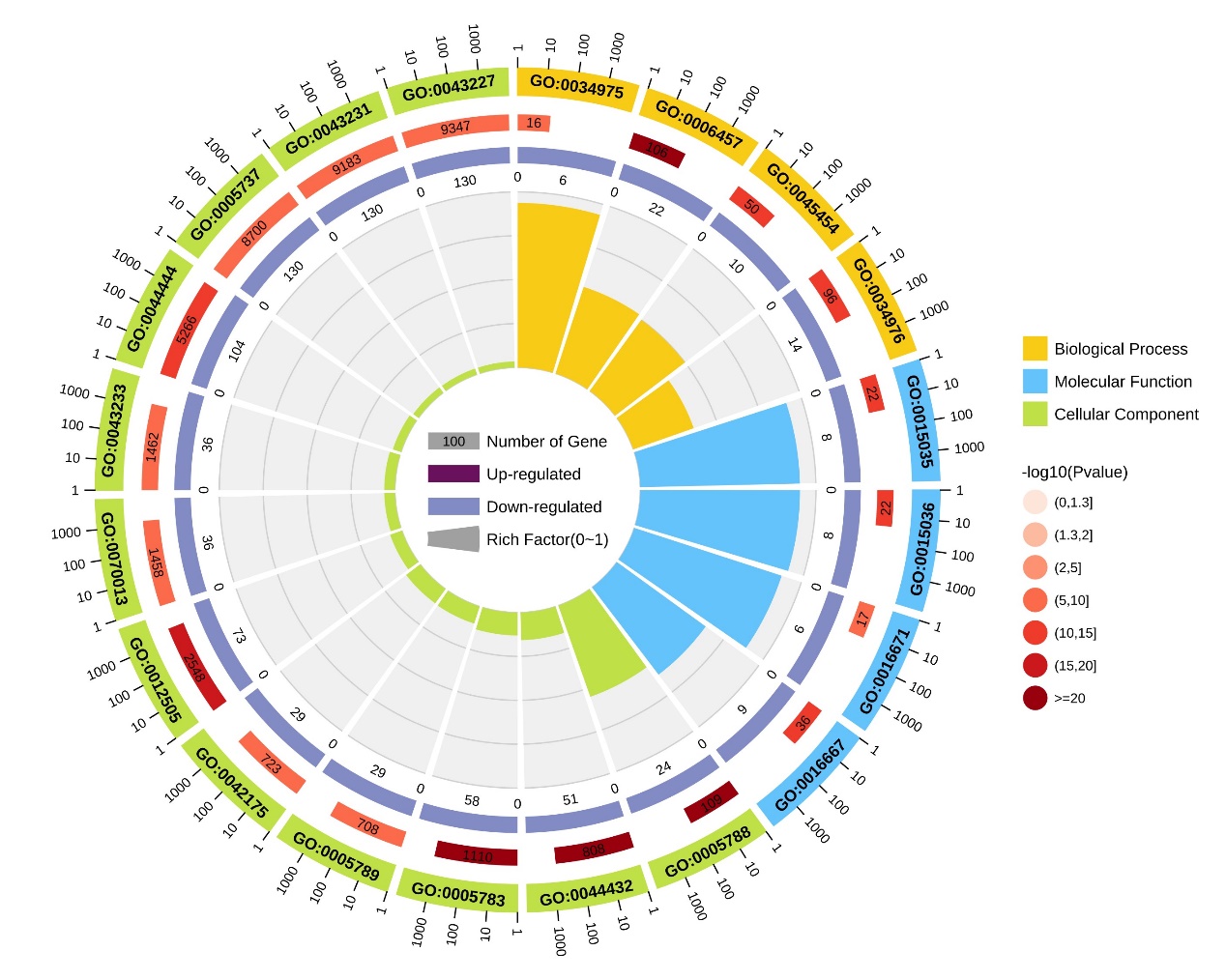


Circle diagram of enrichment of down-regulated genes GO in MidLi vs. HighLi.


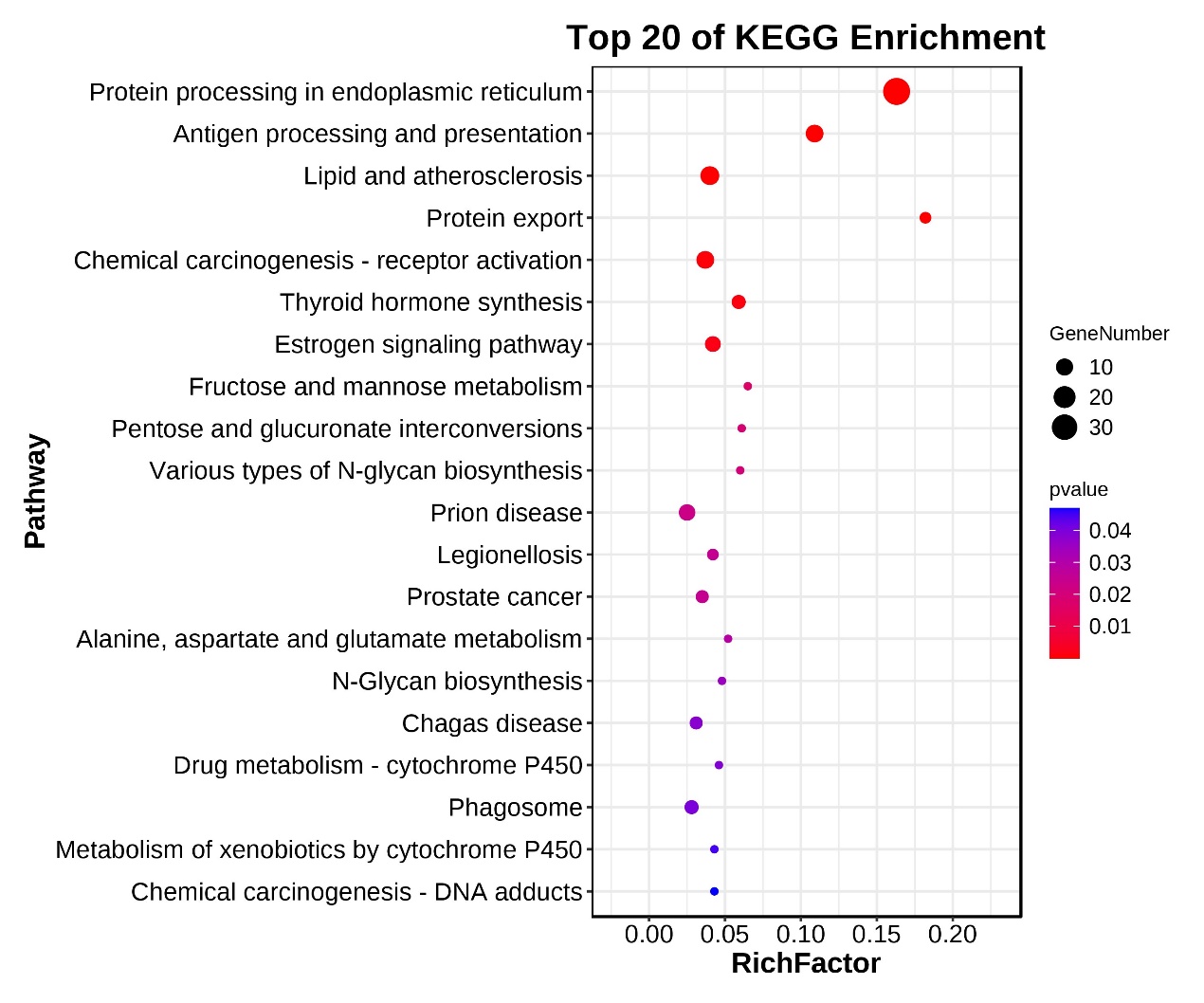


KEGG pathway enrichment scatter plot for MidLi vs. HighLi down-regulated genes.


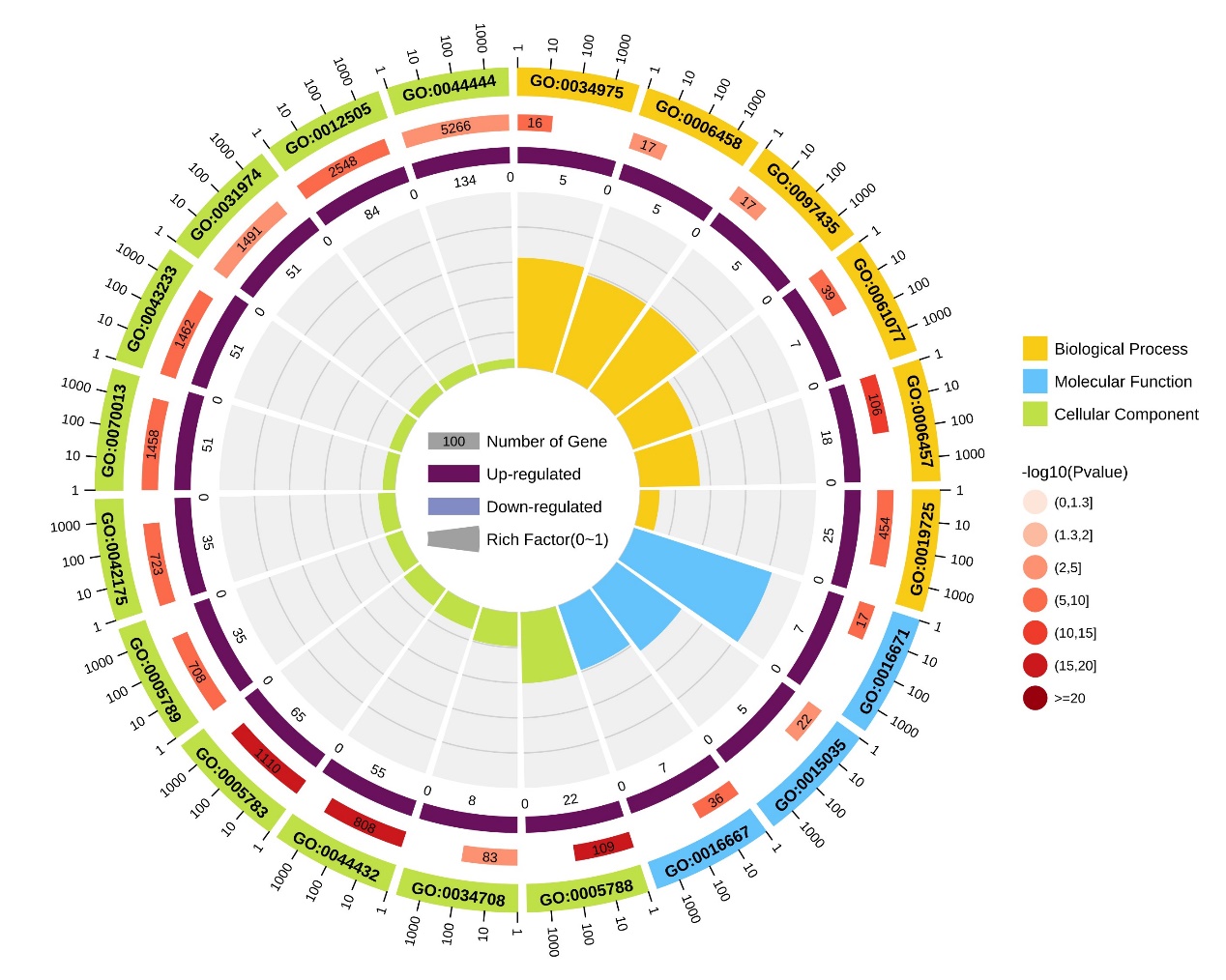


Circle diagram of enrichment of up-regulated genes GO in LowLi vs. HighLi.


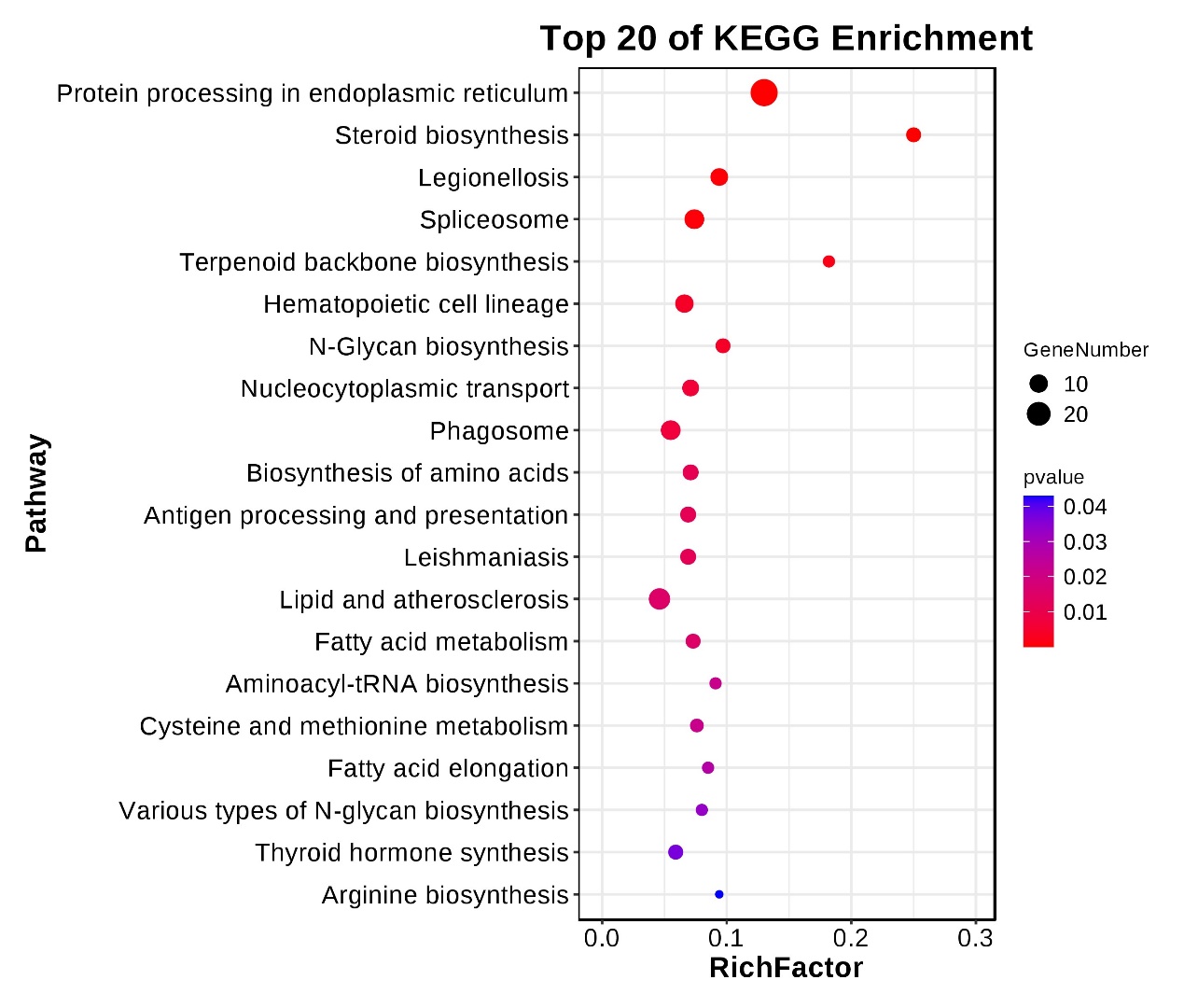


KEGG pathway enrichment scatter plot for LowLi vs. HighLi up-regulated genes.


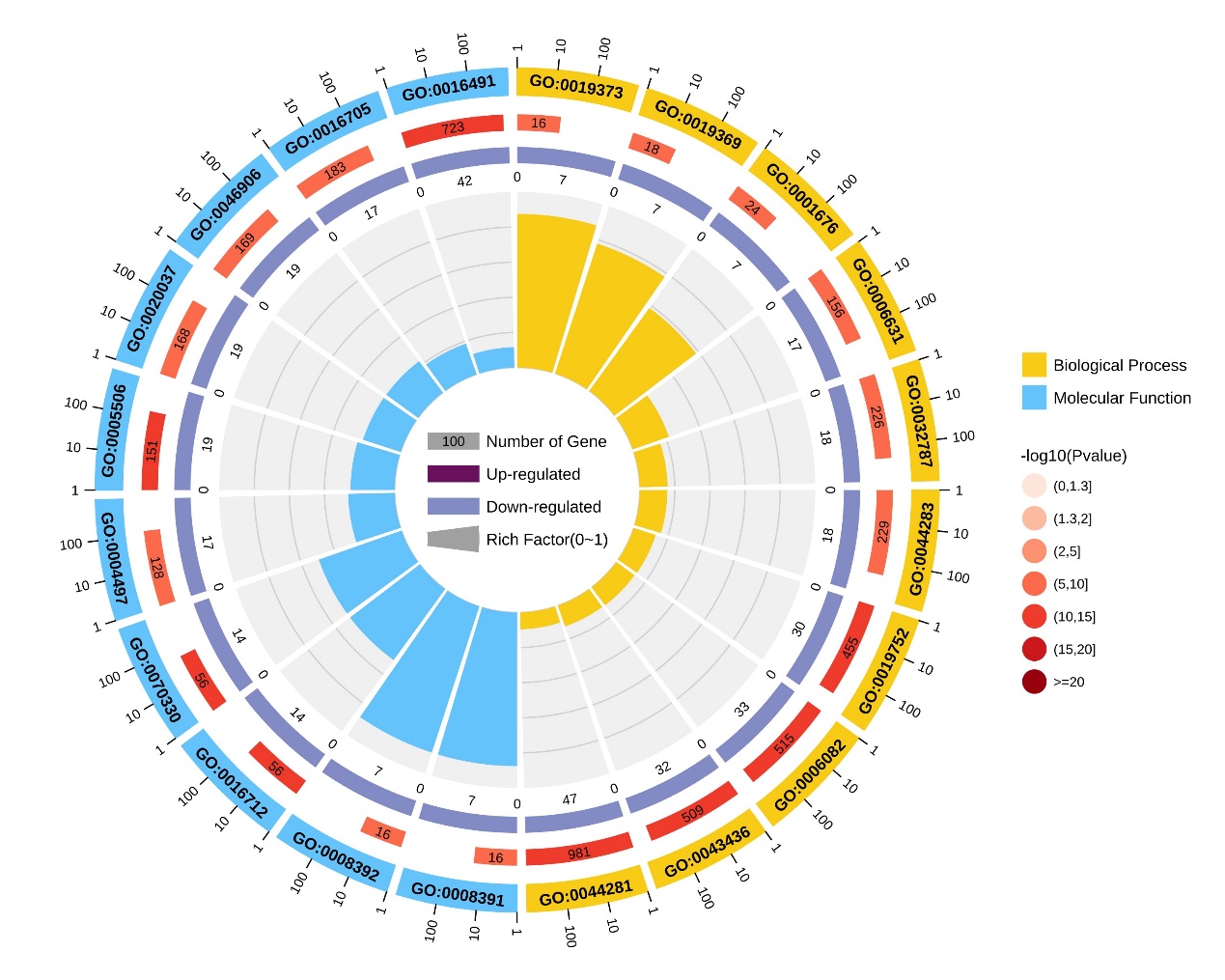


Circle diagram of enrichment of down-regulated genes GO in LowLi vs. HighLi.


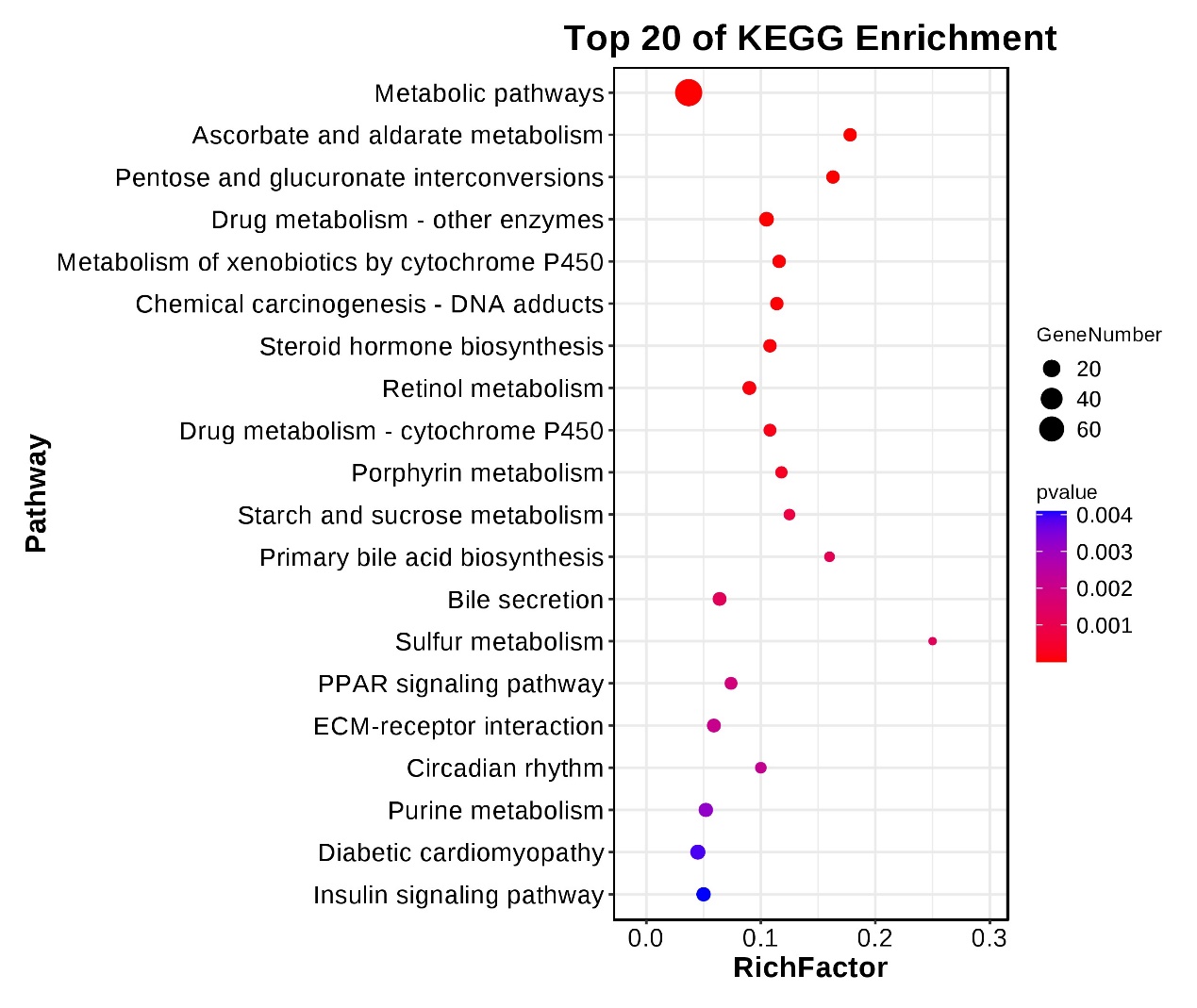


KEGG pathway enrichment scatter plot for LowLi vs. HighLi down-regulated genes.

**miRNA**

**
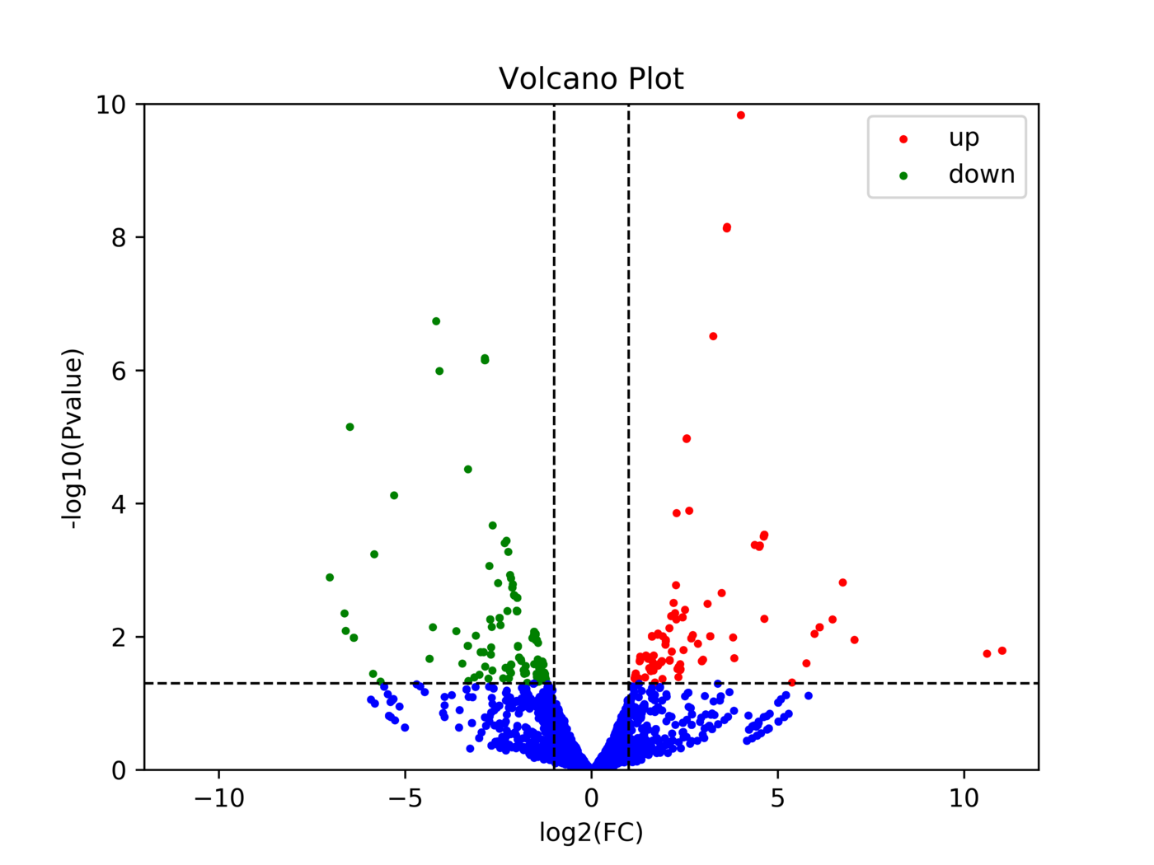
**

Differential expression miRNA volcano plot in LowLi vs. MidLi.

**
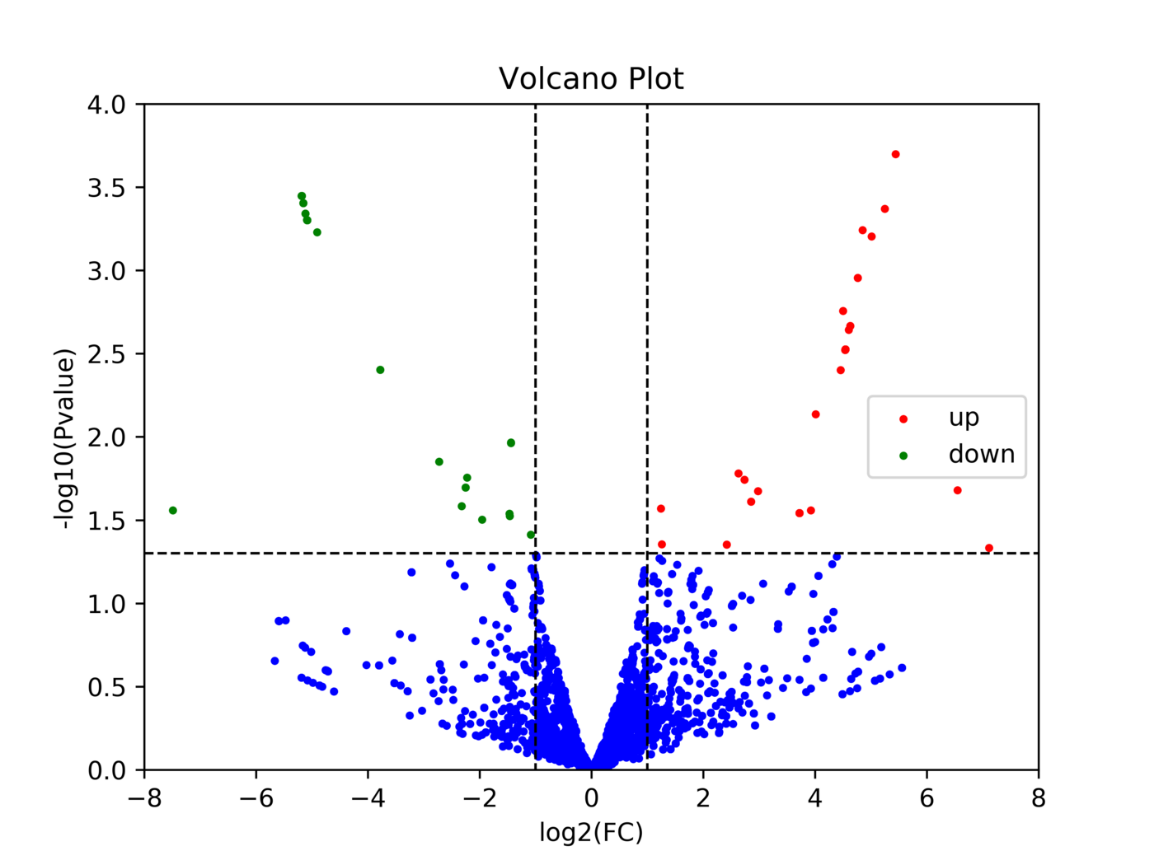
**

Differential expression miRNA volcano plot in MidLi vs. HighLi.

**
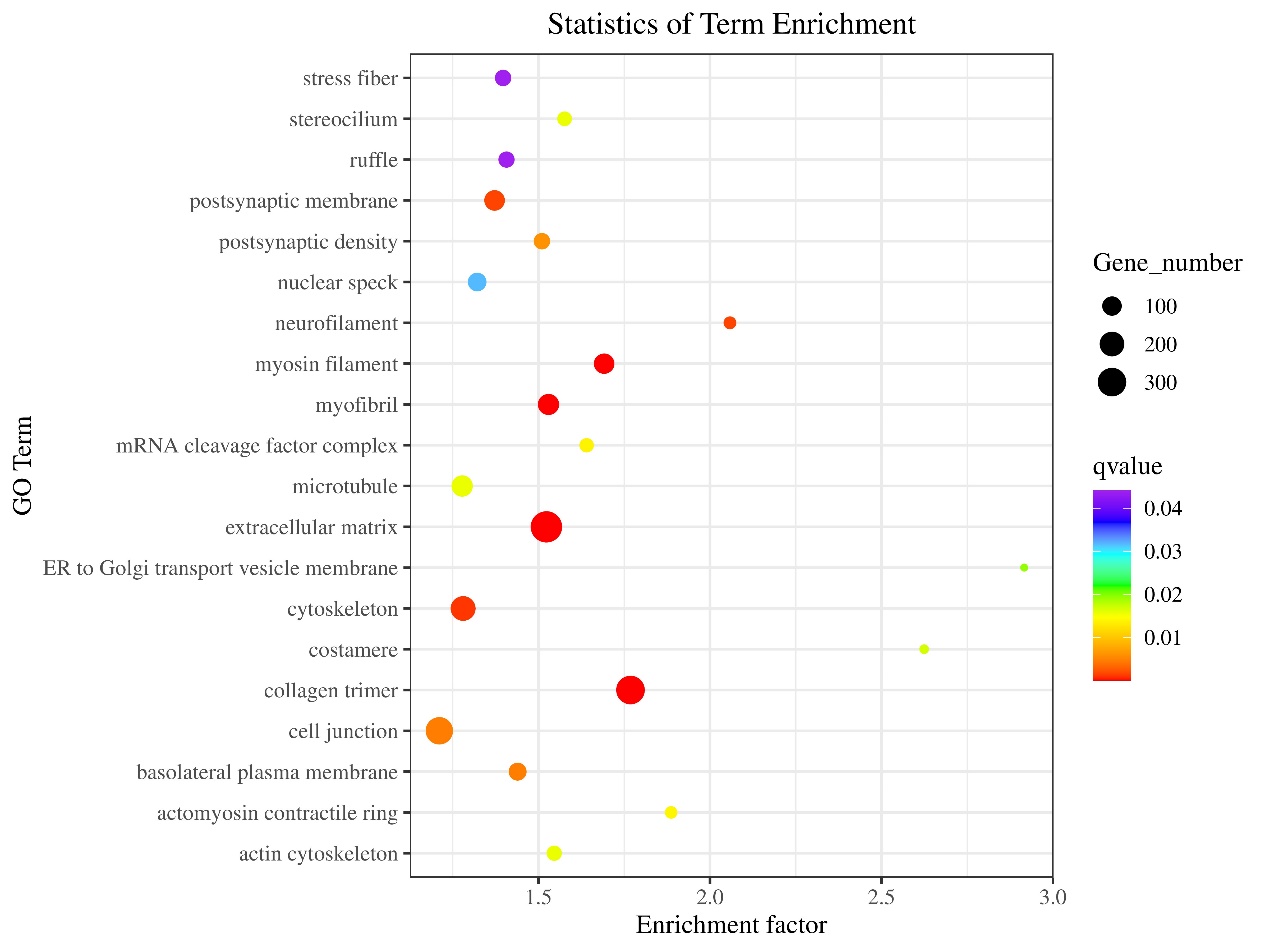
**

Scatter plot of differentially expressed miRNA target genes GO pathway enrichment in LowLi vs. HighLi (cellular components).


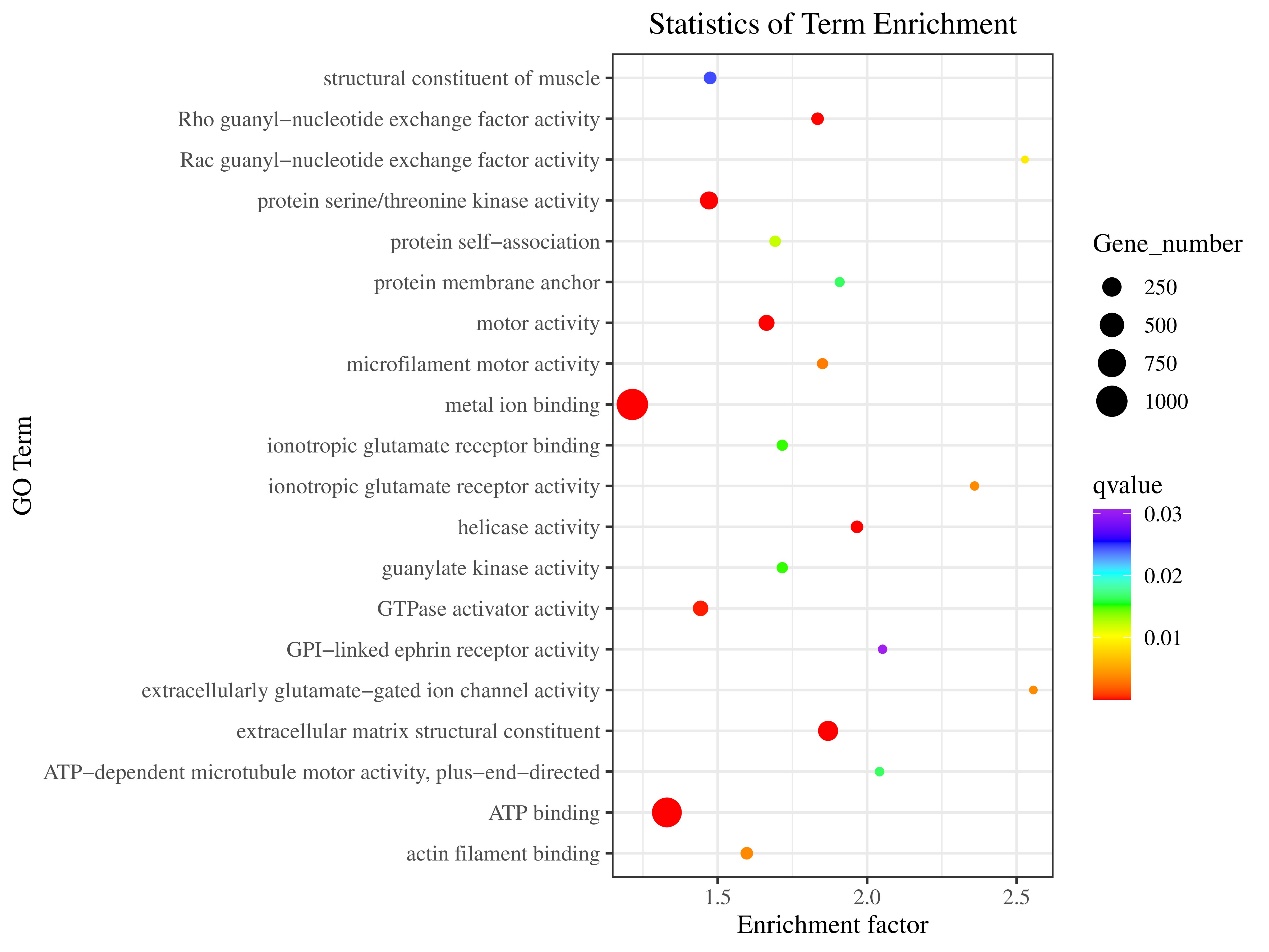


Scatter plot of differentially expressed miRNA target genes GO pathway enrichment in LowLi vs. HighLi (molecular function).


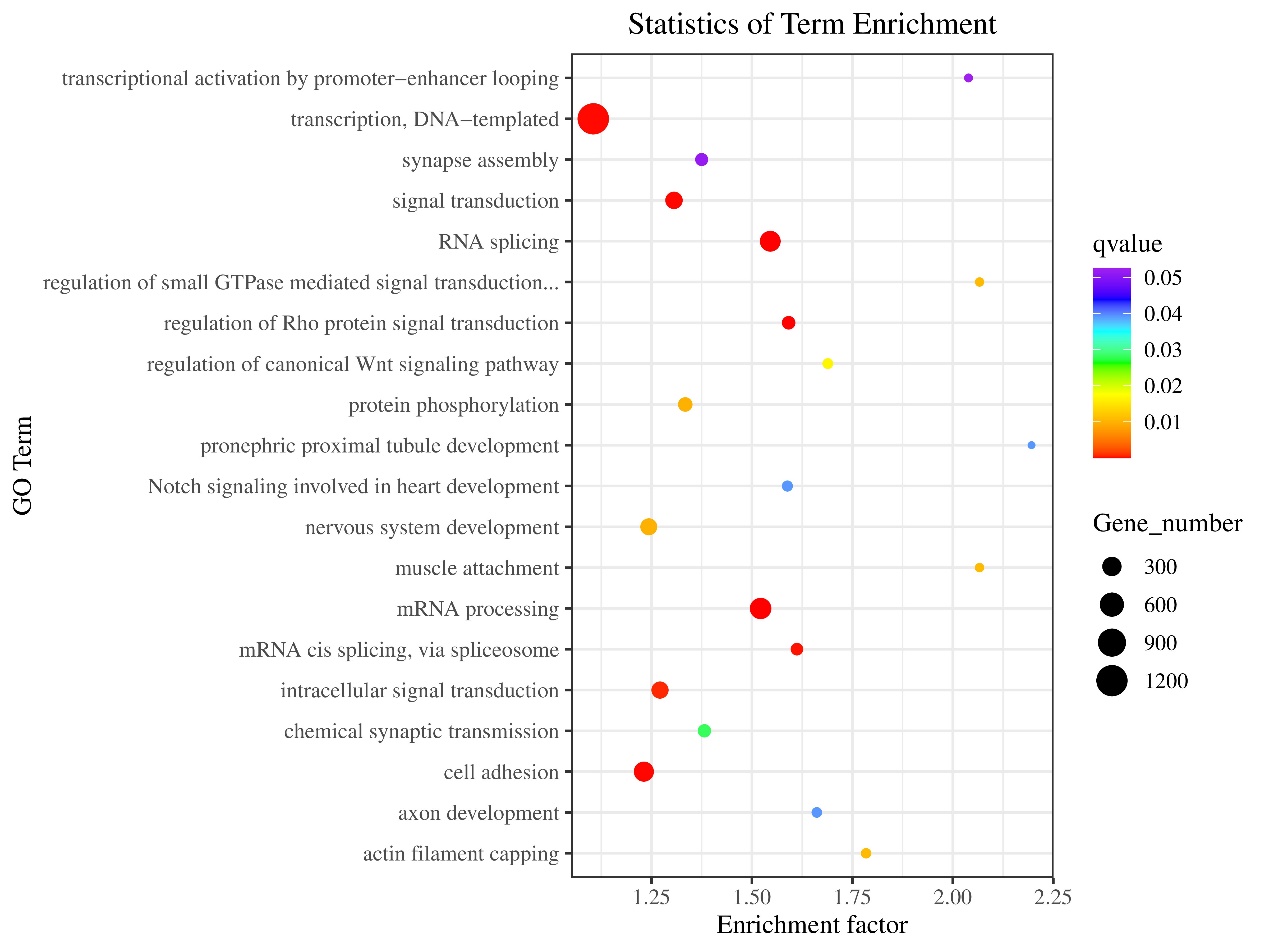


Scatter plot of differentially expressed miRNA target genes GO pathway enrichment in LowLi vs. MidLi (biological process).


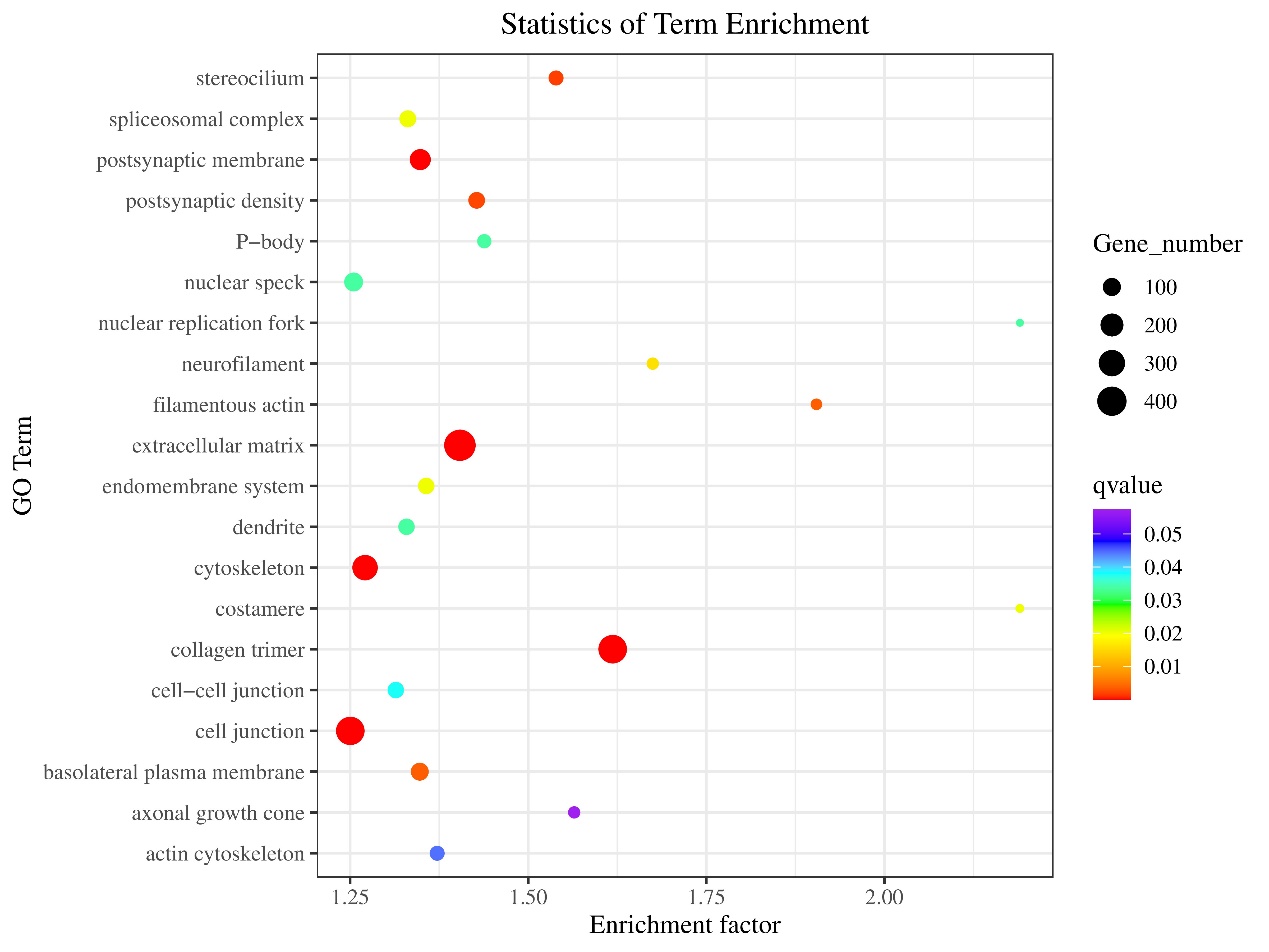


Scatter plot of differentially expressed miRNA target genes GO pathway enrichment in LowLi vs. MidLi (cellular components).


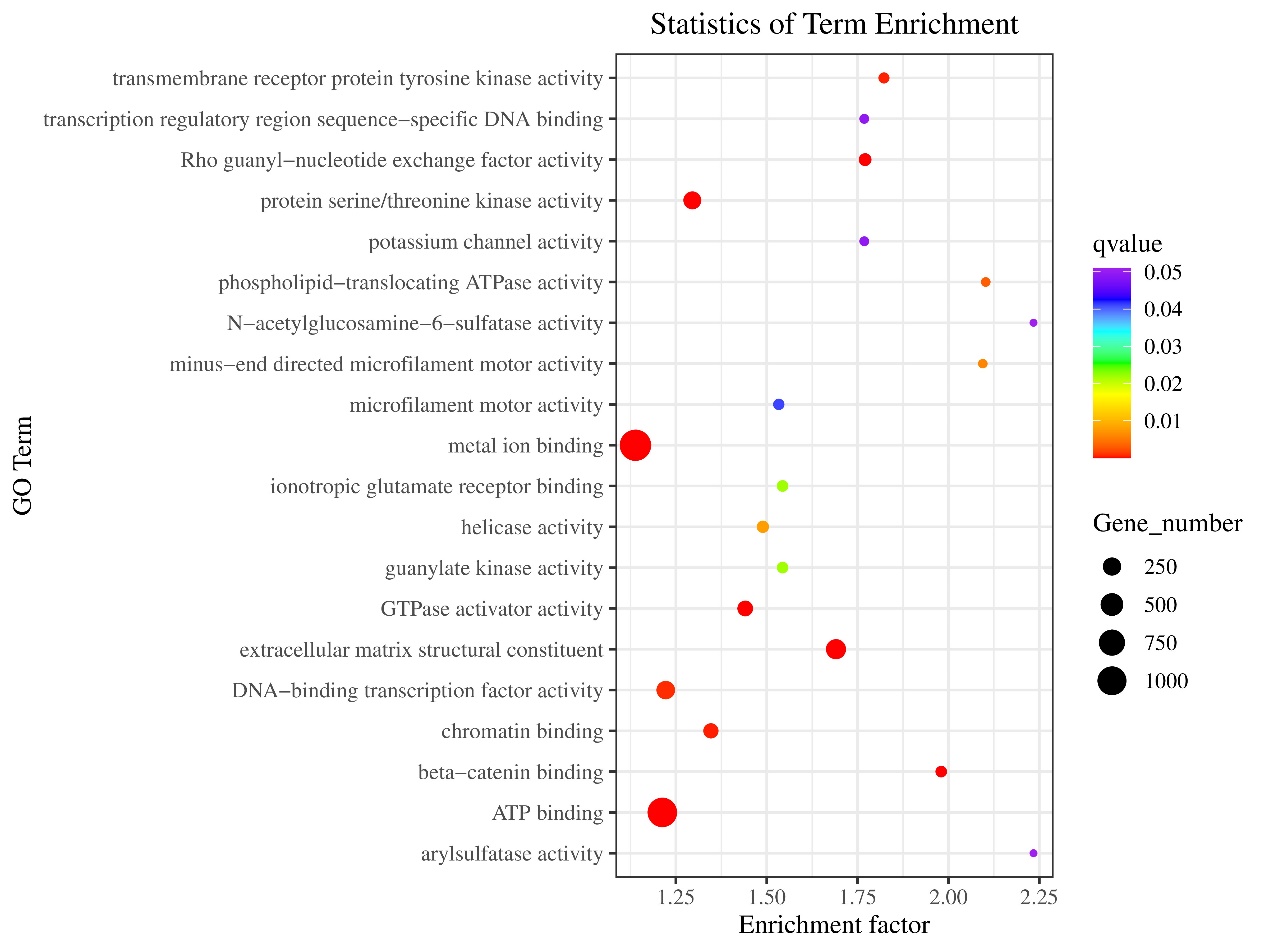


Scatter plot of differentially expressed miRNA target genes GO pathway enrichment in LowLi vs. MidLi (molecular function).


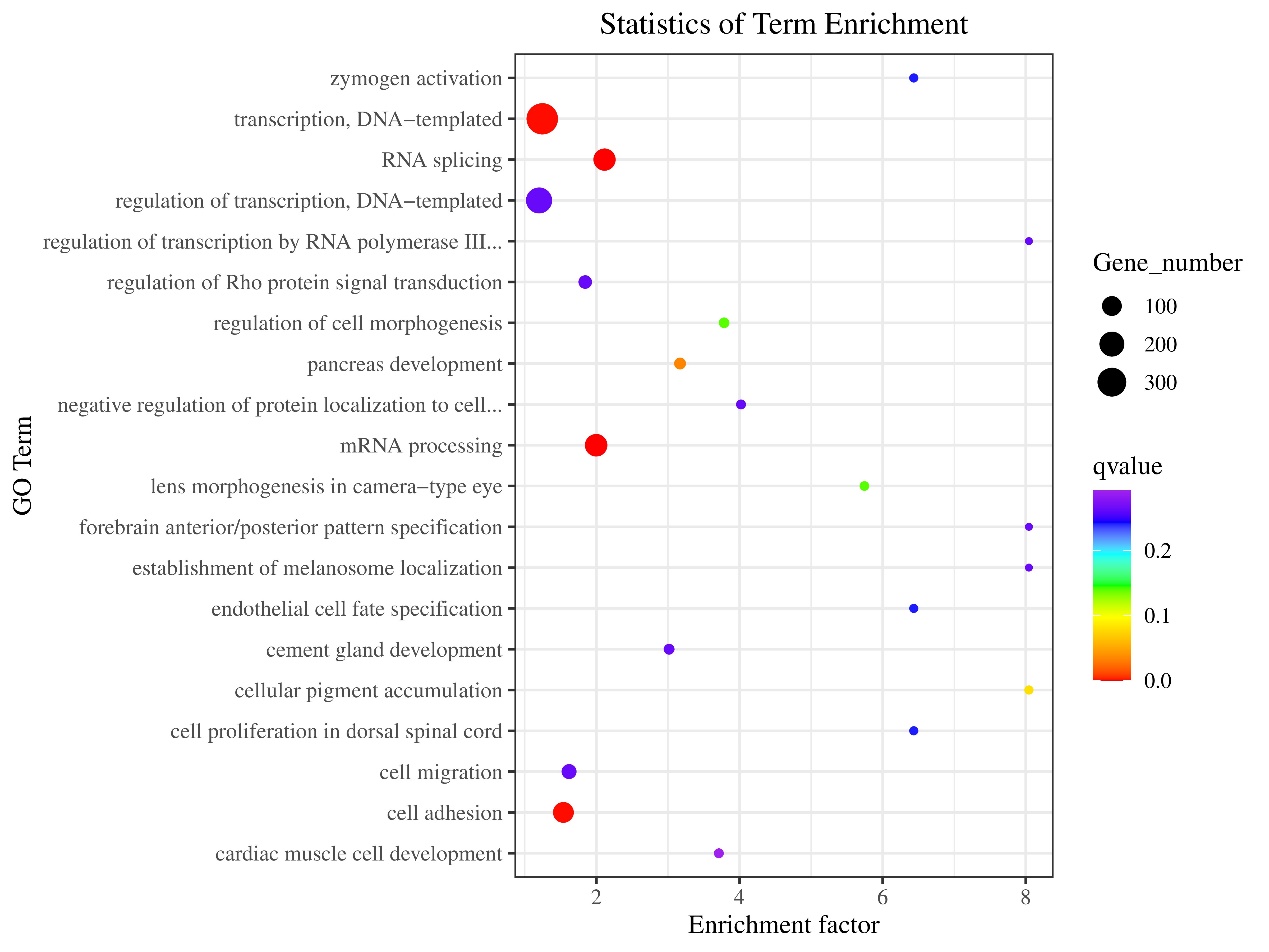


Scatter plot of differentially expressed miRNA target genes GO pathway enrichment in MidLi vs. HighLi (biological process).


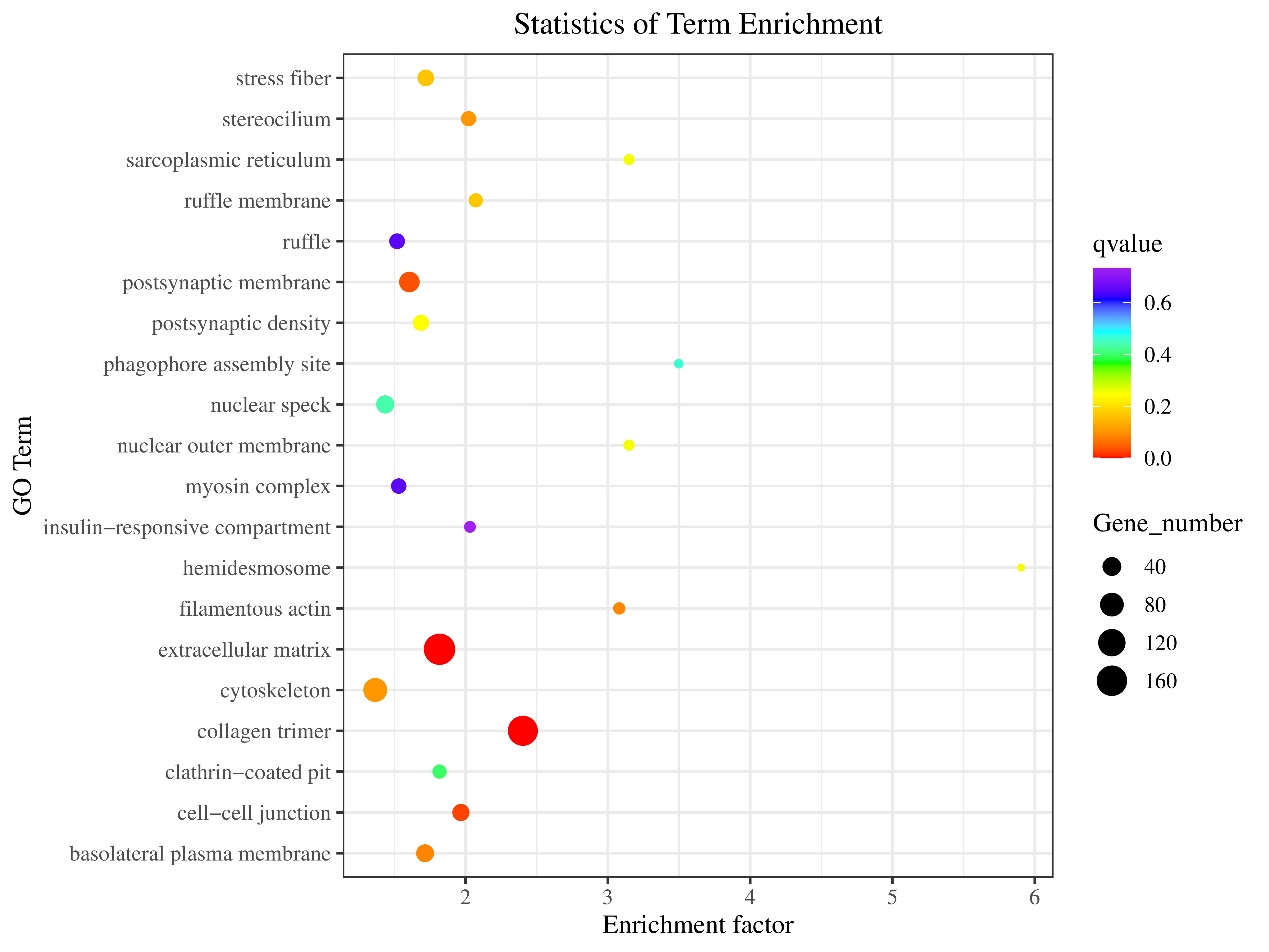


Scatter plot of differentially expressed miRNA target genes GO pathway enrichment in MidLi vs. HighLi (cellular components).


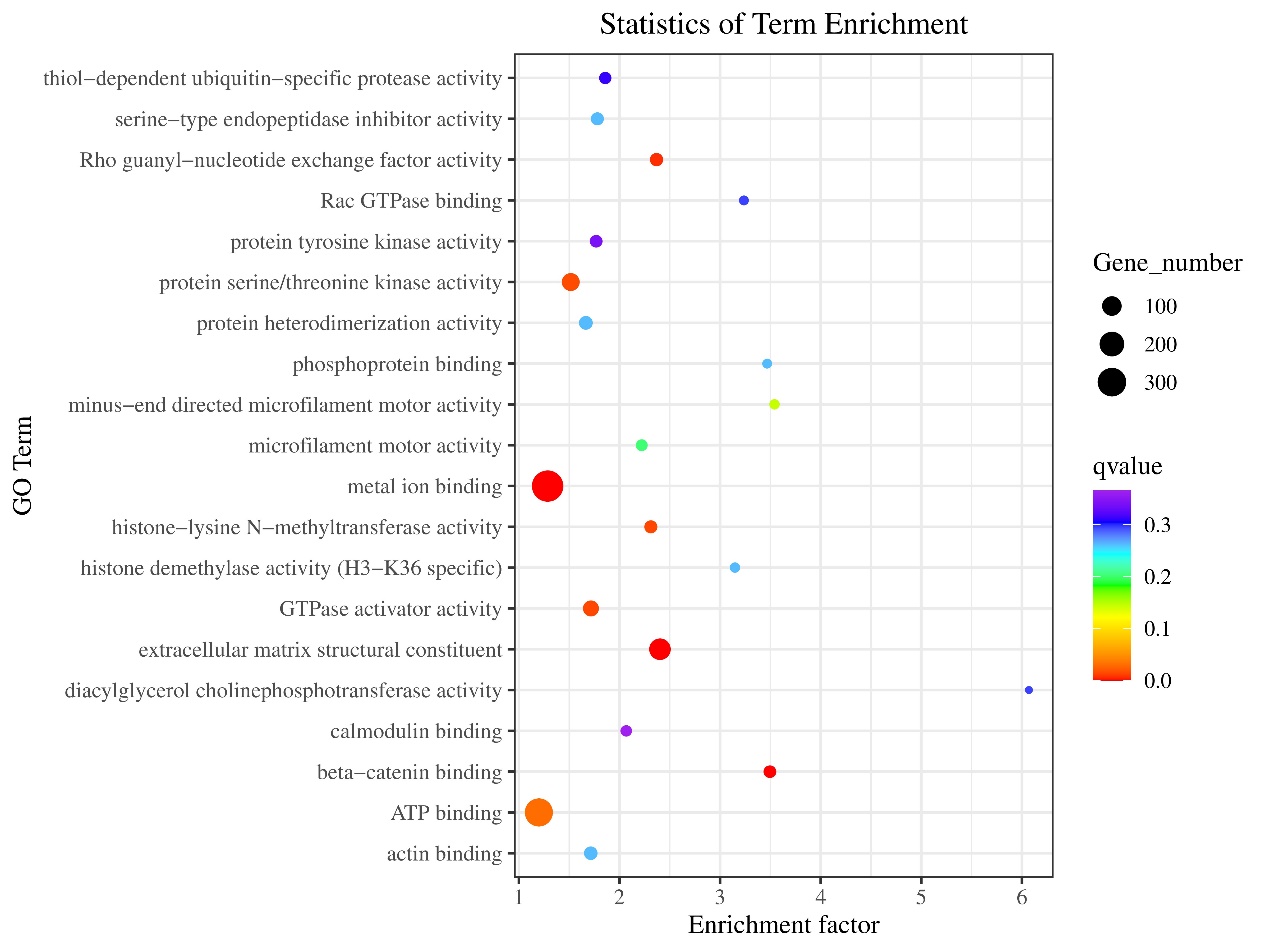


Scatter plot of differentially expressed miRNA target genes GO pathway enrichment in MidLi vs. HighLi (molecular function).


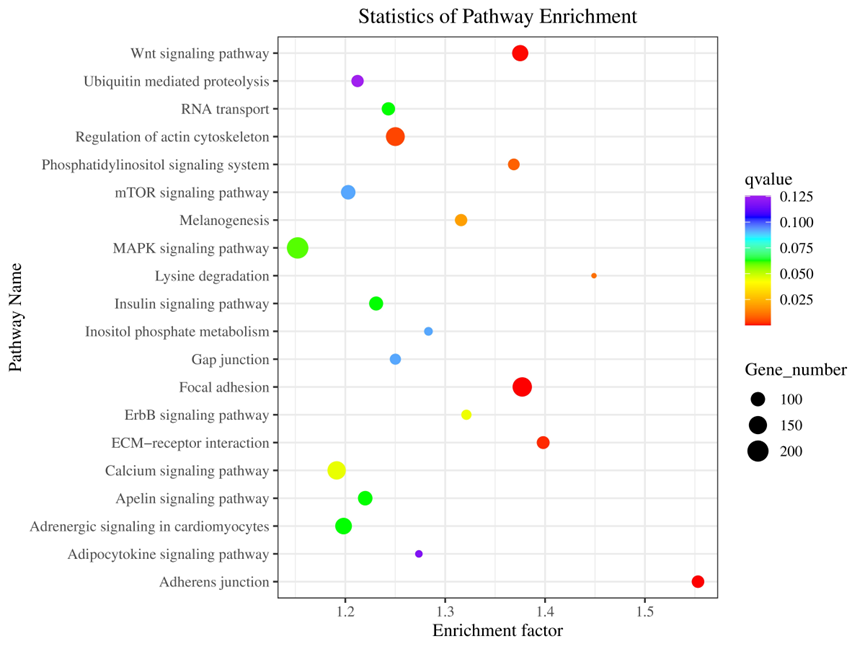


KEGG pathway enrichment scatter plot of differentially expressed miRNA target genes in LowLi vs. MidLi.


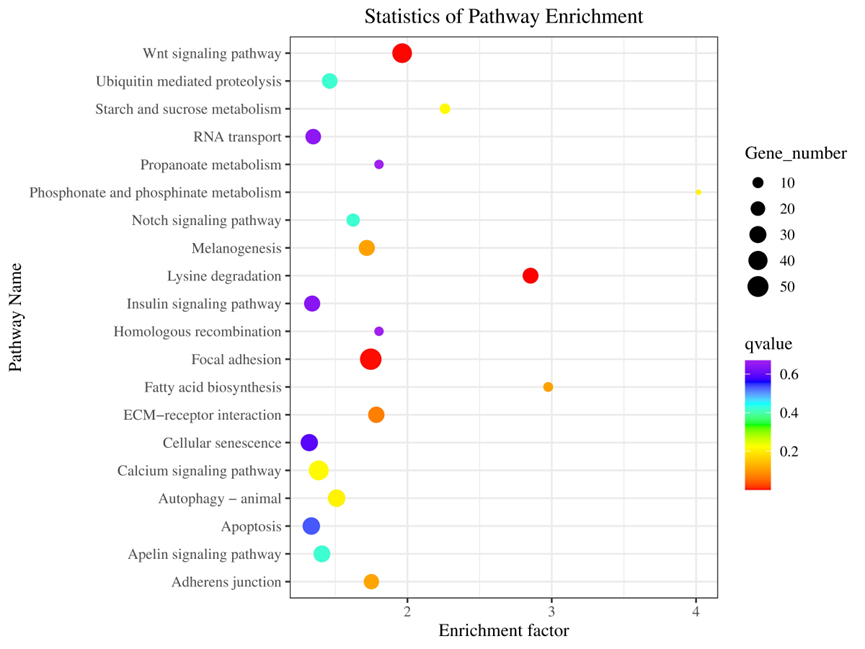


KEGG pathway enrichment scatter plot of differentially expressed miRNA target genes in MidLi vs. HighLi.


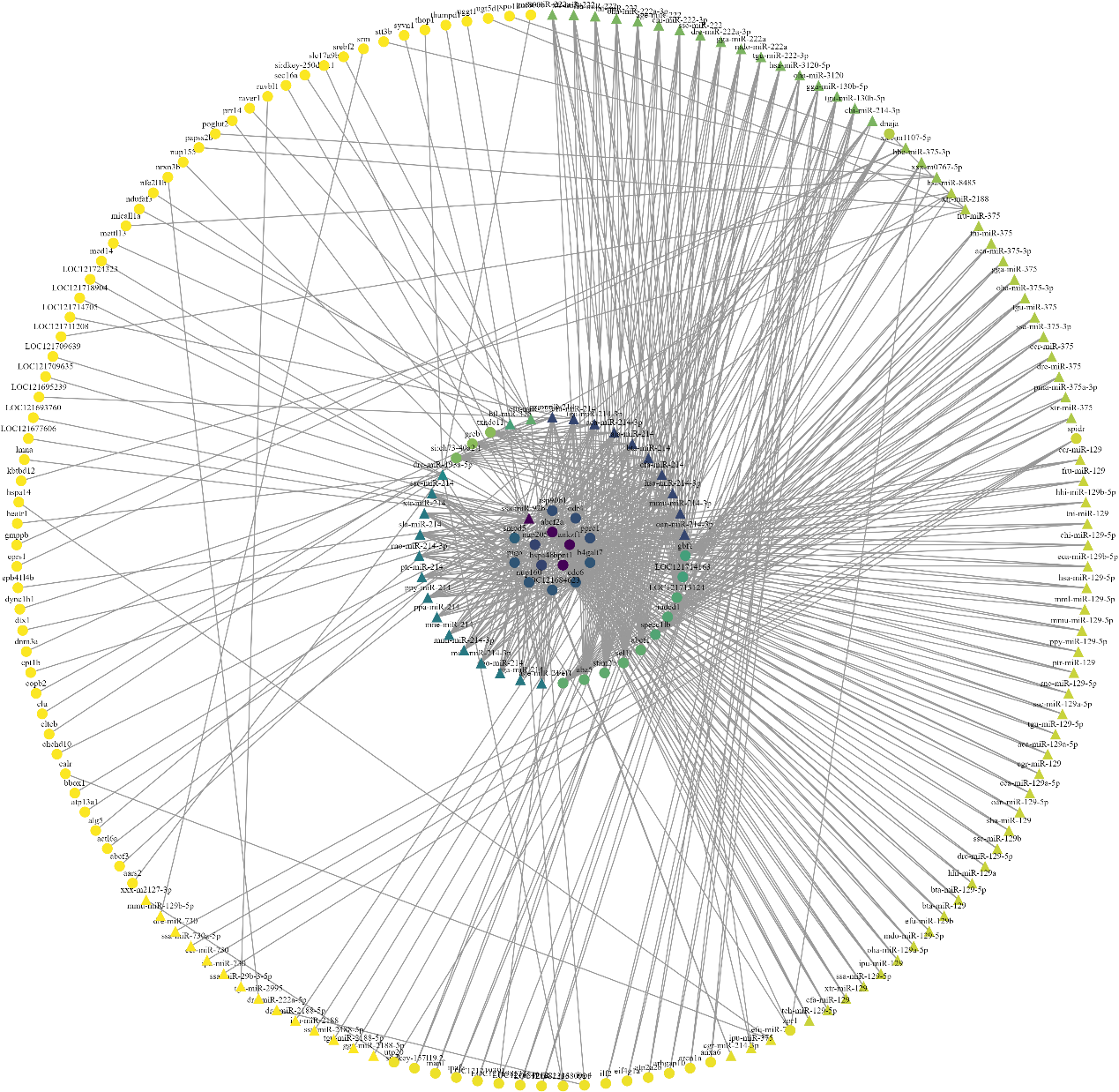


Network diagram of down miRNA-up gene interactions in LowLi vs. MidLi group.


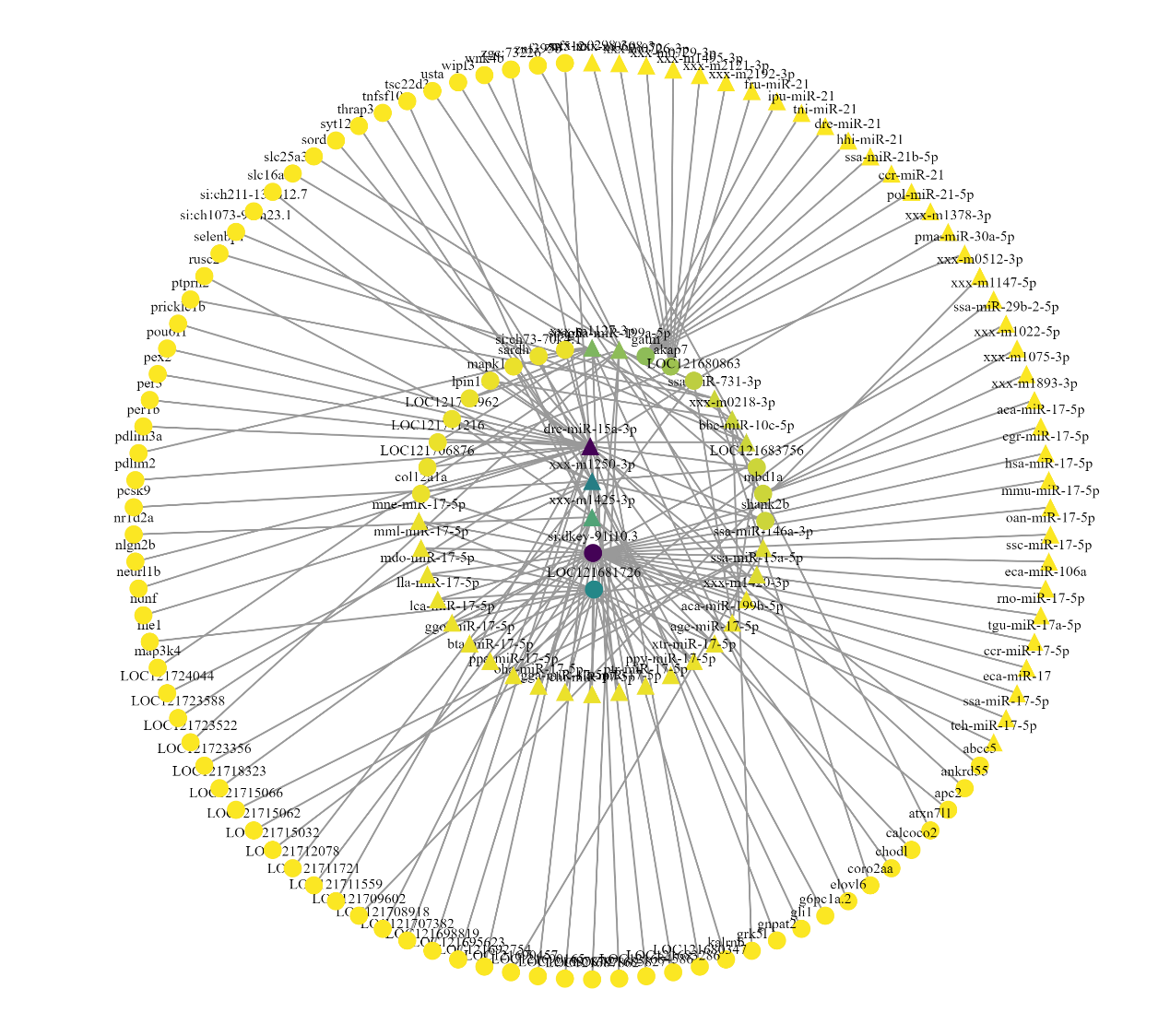


Network diagram of down miRNA-up gene interactions in MidLi vs. HighLi group.


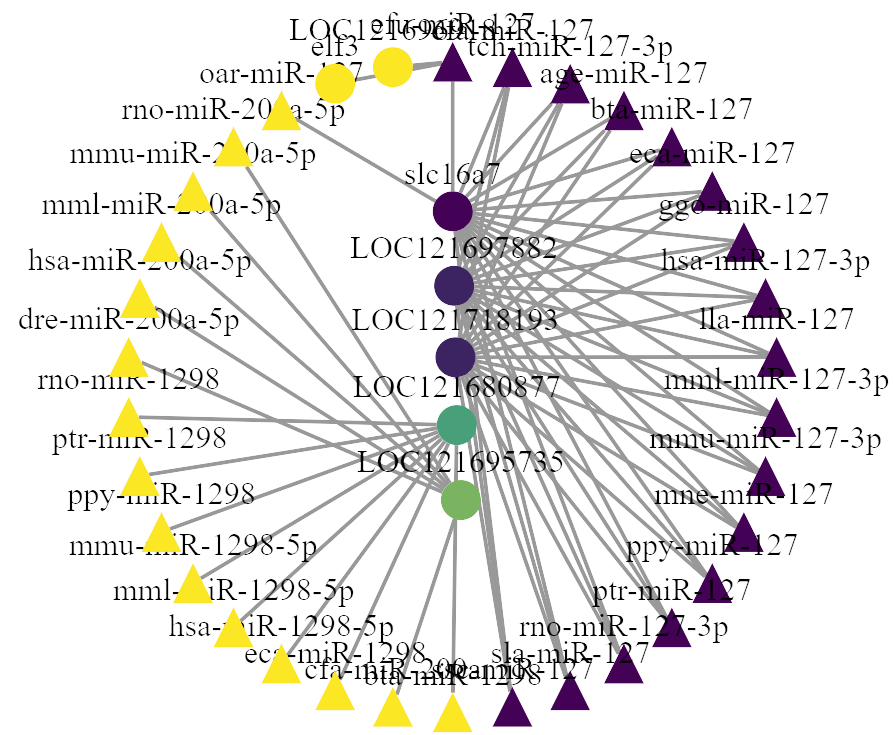


Network diagram of up miRNA-down gene interactions in LowLi vs. MidLi group.
